# Media use among children with ASD: Perspectives and concerns of parents

**DOI:** 10.1371/journal.pone.0332504

**Published:** 2025-10-13

**Authors:** Larissa Pliska, Olga Kunina-Habenicht, Ute Ritterfeld

**Affiliations:** Department of Rehabilitations Sciences, TU Dortmund University, Dortmund, North Rhine-Westphalia, Germany; Father Muller Charitable Institutions, INDIA

## Abstract

Digital media is a significant part of daily life for both adults and children, raising concerns among parents about its impact on child development, particularly for those with autism spectrum disorder (ASD). This study explores the differing perspectives and concerns of parents with and without ASD regarding their children’s media use. A total of 117 parents of children with ASD and 58 parents of typically developing (TD) children participated in an online survey. The study employed group comparisons, correlations, and hierarchical regressions. Results show that parents of children with ASD expressed greater concern about media use compared to TD parents, especially regarding potential negative effects on health and behavior. However, these concerns did not extend to child development or intensification of ASD symptoms. Key predictors of parental concern included challenges in limiting media use, perceptions of media preference and addiction, and children’s ability to cope without media. While parents of children with ASD have notable concerns about media use, these are not excessively pronounced compared to TD parents. Many report allowing digital media as a means for self-regulation in their children. Future research should also examine positive aspects of digital media usage as potential influencing factors.

## Introduction

Children today are growing up with media devices as a matter of course [1,2]. The results of a representative study on media use among 6–13-year-olds in Germany in 2022 show that children are growing up with a broad media repertoire [3]. Almost all households have a TV, smartphone, computer/laptop, and internet access. Fifty-five percent own a tablet, and 51% own a game console. According to parents, children themselves own relatively few devices, but this increases with age. For example, only 9% of 6–7-year-olds own a smartphone, whereas 27% of 8–9-year-olds and more than half (58%) of 10–11-year-olds own a smartphone [3].

As the use of digital technologies continues to grow, the duration of children’s use has become an important issue for concern [4], especially for children with autism spectrum disorder (ASD). This is because ASD is a neurodevelopmental disorder (DSM-5; [5]), and screens are assumed to have a significant impact on children’s neurological development [6]. In addition, people with ASD tend to spend more time in front of screens each day and are at greater risk for screen addiction than people without ASD are [7]. Studies have consistently shown that screen-based media is a preferred leisure activity for children and adolescents with ASD but have reported mixed results regarding whether children with ASD spend more time with screen-based media compared to children without ASD (see the systematic review by Stiller & Mößle [8]). A study by Dong et al. [9] showed that children with ASD spend significantly more time in front of screens (3.34 ± 2.64 h) than typically developing (TD) children (0.91 ± 0.93 h). Other studies also have shown that children with ASD are exposed to more screen time and that this exposure starts at a younger age than TD children [10–12]. These group differences can be found not only in comparison with TD individuals but also in comparison with other clinical groups such as as delayed language development [11].

However, several studies have also reported contradictory results. For example, Mosa et al. [6] reported no significant differences between children with and without ASD in terms of early screen exposure. Additionally, a U.S. national survey of parents of school-aged children (aged 6–17) revealed no difference in screen time between children with and without ASD (TD: 3.21 hours per day; ASD: 3.46 hours per day), where both groups had high screen use [13]. In Germany, Pliska et al. [14] also reported no differences in media use between children (aged 6–11) with and without ASD. Additionally, no differences were found in the frequency of media use or the reasons for use. However, parents of children with ASD reported greater difficulty in limiting their child’s media use than did parents of TD children [14]. Limiting screen time is the most common strategy for regulating children’s use of digital technology [4]. Kuo et al. [15] showed that parents use similar strategies for controlling screen time for adolescents with ASD as they do for their siblings without ASD but more often use more restrictive strategies. Restrictive control is associated with parental concerns about the duration of media use [15]. Overall, parental concerns about media use are significantly related to mediation strategies for adolescents with ASD [15].

### Parents’ beliefs about their children’s media use

Each parent has beliefs (convictions and personal opinions) about children’s media use, which influence how children interact with and grow up with media [3,16]. They have beliefs about the benefits or harms of media or the age at which children should use it [16]. Overall, children spend more time with electronic devices during sensitive developmental periods, such as the first years of life [17]. Some parents are concerned about their young child’s media use [18] while others are not particularly concerned [19]. As a result of being worried, some parents have established rules for the whole family, and others have established rules only for their young children [4]. A qualitative study by Bartau-Rojas et al. [20] indicated that parents share a pessimistic (70.55%) rather than an optimistic (29.45%) attitude toward internet use among primary school children. Some parents are concerned about excessive online time, the ability to handle content, negative effects on learning and academic performance, physical development, social skills and peer interaction, child well-being [20], and potential risks and security threats in virtual environments [21]. In addition, many parents are worried about losing control over the online behavior of their children [20]. Other potential negative effects highlighted by parents include health, psychological development, family relationships, and the security of personal information when social media is used [21]. For example, one negative consequence of screen media consumption is obesity, although there is also indication that the usage of interactive media, such as exergames, can even prevent or reduce obesity [22]. Many parents reported that conflicts with their children about technology use negatively affected their relationships [21]. In this context, a panel study by Matthes et al. [23] revealed that excessive smartphone use by parents was associated with a loss of control over children’s smartphone use, which in turn led to conflicts over smartphone use within the family over time. Overall, parents with a lower education degree tend to have more negative attitudes toward technology than parents with a bachelor’s degree [21]. These negative attitudes were moderate and stemmed from concerns that excessive use could lead to their children becoming dependent on technology or being exposed to inappropriate content [21]. However, parents also have positive views about the impact of media usage, for example, on entertainment, communication, learning, and skill development [20]. In addition, more positive parental beliefs about screen media are significantly associated with intensive sharing and greater consumption of media content perceived as educational [24].

Common concerns of parents can be exacerbated when their child is diagnosed with ASD [25]. Compared with parents of TD individuals, parents of individuals with ASD are significantly more likely to report that the use of electronic devices negatively impacts their children’s behavior [26]. Laurie et al. [25] found that parents of children with ASD had concerns about technology use, especially the amount of time the device was used and the social consequences. Parents’ reported concerns about the use of technology by their children with ASD were related to a longer duration of use. However, it is possible that parents who are very concerned overestimate the time, whereas parents who are less concerned underestimate it [25]. Additionally, children with ASD exhibit clinical characteristics that can contribute to problematic media use, such as deficits in social communication, a tendency to play alone, restricted interests, sensory differences, and difficulties with executive function [27]. They are attracted to interactive media and digital games for longer periods of time because they satisfy their sensory needs and allow them to avoid unpredictable social stimuli without complex control mechanisms [27]. The link between problematic screen use and ASD is likely because screen media meet both sensory stimulation and solitary activity needs, which are both linked to ASD [27,28]. Devices (e.g., smartphones) are often used by children with ASD to provide comfort and help them deal with social overstimulation [29]. Non-functional use of technology, such as repeatedly watching the same video, could play an important role in the lives of children with ASD, for example, as a means of relaxation [25]. New media opens new opportunities for people with ASD, as the internet provides a living space free from the stress of face-to-face encounters [30]. Additionally, individuals with ASD can modify their communication environment via technology, for example, by using asynchronous options and reducing the use of sensory stimuli to meet different social needs [31]. Shane and Albert [32] show that, when given more free time, such as on weekends, children with ASD tended to engage in media interaction instead of other play activities, in comparison to when they had less free time, such as during the week. Parents also report significant verbal and physical imitation during and after electronic screen media use [32]. Media is often seen as a tool to improve communication skills of children with ASD [33]. In addition, more than half of the parents reported positive effects of media use on their children’s emotional regulation. A significant correlation was found between time spent in front of television or the internet and the ability to cope with stress [33]. However, parents use media in part to regulate their child’s distress [34] and manage their child’s difficult emotions, especially if the child has a more challenging temperament [35]. The results of an analysis of media use for emotion regulation in young children (2–3 years) revealed that higher levels of media emotion regulation were associated with more problematic media use and more extreme emotions when the media was removed [35].

Overall, the relationships among parental beliefs, total screen time, use for behavioral regulation, and limit setting depend on parental stress levels and, to a lesser extent, parental media skills [24]. In other words, the relationships between parental beliefs and screen media use practices are less pronounced for parents with lower media skills and higher stress levels [24].

### Screen time and ASD

Recently, research on the effects of screen time on children with ASD has increased due to a growing interest in the causes of ASD symptoms and therapeutic approaches that incorporate technology and electronic devices [36]. Alrahili et al. [17] found a significant correlation between digital device use and deficits in the development of social skills and symptoms indicative of ASD. Research indicates that greater screen exposure in children is associated with a greater likelihood of an ASD diagnosis [7,37], with those in the highest exposure group having a 97% greater chance of a diagnosis than those in the lowest exposure group [7]. Digital media use before 21 months of age was associated with ASD risk (sensitivity of 71% and specificity of 72%), and the risk increased when mothers spent less than 6.5 hours per day with their child [12]. Hill et al. [38] showed that children who were later diagnosed with ASD or attention-deficit/hyperactivity disorder (ADHD) had, on average, more than twice as much screen time as the comparison group, and the ASD and ADHD groups had similar screen time. Increased screen time at 18 months of age was significantly associated with greater symptoms of ASD and ADHD, as well as lower developmental scores at 3 to 5 years of age. The authors noted that increased screen time, along with the challenges associated with ASD and ADHD, may have a cumulative effect on development. However, they emphasize that their study cannot determine whether increased screen time at an earlier stage of development leads to increased neurodevelopmental symptoms or is secondary to other factors associated with developmental trajectories and behavioral phenotypes [38].

A systematic review and meta-analysis of the association between screen time and ASD suggests that the purported link is not well supported by the existing literature, although the observed effects are most pronounced in children [39]. However, the direct effect of excessive screen time during early childhood on the increased risk of ASD remains inconclusive, with some studies reporting an association (e.g., [11,40]), whereas others finding no association (e.g., [13]).

### Virtual autism

The concept of “virtual autism” was first introduced by Marius Zamfir, who hypothesized that daily screen time in excess of four hours or more could induce sensory-motor and socio-emotional deprivation in children 0–3 years of age, similar to the behaviors of children with ASD [41]. Several studies have confirmed the presence of ASD-like symptoms in young children overexposed to electronic screens [42]. For example, research indicates that excessive screen time in young children is associated with developmental and behavioral issues resembling ASD, including language delays, social challenges, emotional instability, and attention problems [43–45]. In addition, a study by Al Moussawi et al. [46] highlighted the strong association between earlier and longer screen use and the development of ASD-like behaviors, with boys showing greater susceptibility to ASD-like traits. A single-case-study [43] and a cross-sectional study [44] further support that longer screen time correlates with developmental delays and ASD-like behaviors, especially when it exceeds two hours daily. Furthermore, a cohort study by Heffler et al. [40] revealed that early screen exposure and less interactive play at 12 months were linked to more ASD-like symptoms at age two, although not necessarily increasing ASD risk. Children with ASD tend to spend more time on screens, with longer screen time being associated with more severe ASD symptoms (particularly sensory symptoms) and more significant developmental delays [9]. This is particularly the case for children with ASD who spend longer times in front of a screen and are younger [9]. An intervention by Heffler et al. [47] demonstrated that replacing screen time with parent-child social engagement significantly reduced screen use from 5.6 hours to 5 minutes daily and improved core ASD symptoms and parental distress over six months. Similarly, a study by Nisar et al. [48] suggested that ASD-like symptoms through excessive screen time in young children are reversed with early intervention. Overall, reducing screen time and enhancing parent-child interaction are essential strategies to mitigate developmental and behavioral issues linked to excessive digital media use in early childhood [43,49].

Although children with virtual autism exhibit behaviors similar to those of children with ASD, distinguishing between the two is important [50]. ASD is a neurodevelopmental disorder that involves genetic, biological, and environmental influences and is typically diagnosed on the basis of developmental markers, whereas virtual autism, which is not an official diagnosis [51], appears to be due primarily to environmental factors, particularly the overuse of digital technologies during critical developmental periods [50]. Studies by Chakraborty [50] and Rakshit and Biswas [52] suggest that virtual autism may be reversed by reducing screen time and increasing participation in non-media activities, whereas ASD requires long-term interventions. The use of terms such as “virtual autism” remains therefore controversial. Critics argue that labeling developmental problems caused by excessive screen use as “autism” is misleading and may lead to a misunderstanding of ASD [42].

### Study aims and research question

The literature provides contradictory evidence regarding parents’ concerns about their children’s media use (see ‘Parents’ beliefs about their children’s media use’). Some parents are concerned [18] while others are not [19]. In addition, parents’ concerns may intensify if their child is diagnosed with ASD [25]. However, contrasting results can also be found among parents of children with ASD (see ‘Parents’ beliefs about their children’s media use’). On the one hand, studies indicate that parents of children with ASD are concerned about the potential negative effects of media use on their children’s behavior [26]. However, other studies highlight the positive impact of media on children with ASD [33]. Overall, it is unclear whether parents’ concerns about media use differ depending on whether or not their child has ASD. One of the aims of this study is therefore to investigate whether the concerns of parents with children with and without ASD differ in relation to their children’s media use. The research question in this regard is: Do parents’ attitudes and concerns differ between children with and without ASD?

Additionally, contradictory results have been reported regarding the potential association between excessive screen time during early childhood and an increased risk of ASD (see ‘Screen time and ASD’). Furthermore, it has been shown that expressive media exposure can cause ASD-like symptoms (see ‘Virtual autism’). Therefore, the study also aims to examine, whether parents’ attitudes and concerns differ regarding media consumption and ASD-like symptoms or ASD development.

## Method

### Participants

In accordance with the Declaration of Helsinki, a positive ethics vote by the Ethics Committee, Department of Rehabilitation Sciences, TU Dortmund University (GEKTUDO-2024–54) was issued. From November 11, 2024, to May 15, 2025, a total of 526 individuals accessed the online parent questionnaire and provided written informed consent, mainly through autism therapy centers, self-help groups, parent groups, social media, and private contacts in Germany. However, 319 forms were discarded because they were incomplete. Of the remaining 207 questionnaires, 27 questionnaires were discarded to reduce the dataset to families with children who were developing typically (no diagnosis or suspicion) or had already been diagnosed with ASD. Five more questionnaires were removed because the children were outside the 6–11 age range. The final dataset consists of 175 completed questionnaires (dropout rate of 66.7%). Of those 117 were assigned to the ASD group and 58 to the TD group.

On average, the children in the ASD group (*n* = 117) were 106.15 months (8;10 years) old (*SD* = 19.7; age range 6;0–11;09). The sex ratio was 4:1 (93 males and 24 females), which is consistent with the prevalence of ASD [53–55], although a ratio of 3:1 is now assumed [56]. Among the 117 children, 35 had only an ASD diagnosis, while 66 also had comorbid AD(H)D. Other comorbidities included intellectual disability, language development disorder, dyslexia, learning disability, and dyscalculia. At an age-appropriate level, communication with others is possible for 88 children, communication with others is possible only to a limited extent for 24 children, and 4 children have no verbal language. In the ASD group, 25 children had no siblings, 55 had one sibling, 30 had two siblings, and seven had three siblings. On average, the children in the TD group (*n* = 58) were 104.84 months (8;08 years) old (*SD* = 16.29; age range 6;03–11;08). The TD group had a sex ratio of 3:2 (36 males and 22 females). Despite the different sex ratios, the two groups can be used for comparison since no gender-specific differences in media usage were found by MacMullin et al. [26]. At an age-appropriate level, communication with others is possible for 57 children, and communication with others is possible only to a limited extent for one child. In the TD group, 12 children had no siblings, 26 children had one sibling, 11 children had two siblings, six children had three siblings, and three children had four to six siblings. For both groups, the questionnaire was predominantly completed by mothers (see Table 1).

**Table 1:** Information about the person who completed the questionnaire.

Information about the person who completed the questionnaire	ASD (*n* = 115; missing = 2)	TD (*n* = 57; missing = 1)
The questionnaire was completed by	• Mother: 109 • Father: 4 • Other person: 2	• Mother: 45 • Father: 10 • Other person: 2
Age	*M* = 39.88 (*SD* = 5.4) Range: 27–52 years	*M* = 41.12 (*SD* = 5.85) Range: 25–57 years
Diagnoses	• No: 68 • Yes: 47 - AD(H)D - ASD - ASD and AD(H)D	• No: 55 • Yes: 2 - AD(H)D
Highest level of education: Person 1 (i.e., mother)^1^	• Less than high school degree: 38 • High school degree: 37 • College degree and more: 39	• Less than high school degree: 4 • High school degree: 14 • College degree and more: 38
Highest level of education: Person 2 (i.e., father)^2^	• Less than high school degree: 47 • High school degree: 23 • College degree and more: 36	• Less than high school degree: 12 • High school degree: 10 • College degree and more: 35

^¹^The person who answered the questionnaire (i.e. mother).

^2^Information provided by the person who completed the questionnaire about the highest level of education of another person/ caregiver (i.e. father).

### Measures

An ad hoc online survey for parents was used to collect data on children’s media usage and parents’ attitudes and concerns about their children’s media usage. Items from different questionnaires were tailored to the research question for this purpose. The final questionnaire can be found in the supporting information, S1 and S2 Appendix. The survey included items about the types of media available at home, the frequency and duration of children’s media usage, and the reasons for using digital media. Wood et al. [57] showed that parents and children generally agreed on their responses to media exposure, allowing parental responses to be used. Parents were asked to provide information about how much time their children spend with digital media during the week and on weekends. They were asked to use the following categories: “Never = 0”, “Up to 30 minutes = 1”, “One hour to two hours = 2”, “Two hours to four hours = 3”, and “More than four hours = 4”. Parents were also asked to state the maximum number of minutes or hours their child should spend with digital media during the week and on weekends. For parents who specified time periods rather than specific times, the mean was calculated. Parents were asked to rate various statements about their child’s use of digital media in everyday life via a Likert scale ranging from 1 (“Never applies”) to 10 (“Almost always applies”). The items on the child’s use of digital media in everyday life were summarized into three subscales (see supporting information, S3 Appendix): preference for digital media and media addiction (15 items, α = .9); media skills (4 items, α = .56); and restrictions and challenges in regulating media use (5 items, α = .82). High scores on the media addiction subscale indicate greater dependence on media. High scores on the media skills subscale indicate greater media skills, whereas high scores on the restrictions and challenges in regulating media use subscale indicate fewer restrictions and greater difficulty in implementing them. Finally, they were asked to rate statements about their thoughts and concerns regarding their child’s media use on a scale from 1 (“Disagree”) to 10 (“Agree completely”). The items regarding parents’ concerns about their children’s media use were summarized into five subscales (see supporting information, S3 Appendix): media addiction (3 items, α = .82); loss of connection to the real world (4 items, α = .89); negative effects on health and behavior (7 items, α = .85); worries about online dangers, loss of control, and parental media skills (8 items, α = .84); and no support as a reason for worries (2 items, α = .78). Here, higher values indicate greater worry. The concern scale without ‘No support as a reason for worry’ subscale, comprising 22 items, has a Cronbach’s alpha of.95. Additionally, the two items related to concerns about media use and ASD symptoms were combined into a subscale with a Cronbach’s alpha of.67. To account for missing values, the subscales were formed based on the mean value.

### Data analysis

Descriptive and statistical data analysis was carried out via R version 4.4.2 [58] (see R code in supporting information, S4 Appendix). Group comparisons, correlations, and hierarchical regressions were performed to address the research question. The ‘psych’ and ‘car’ packages were used for descriptive data and to check the assumptions. The Shapiro test was used to calculate the normal distribution of the variables, and the Levene test was used to calculate the variance homogeneity. If these prerequisites were violated, the Mann-Whitney *U*-test was used as a non-parametric alternative for group comparisons and Spearman’s correlation, both of which were corrected via the Bonferroni method. For significant results in the Mann-Whitney *U*-test, the effect sizes (rank correlation coefficient = *r*) were also calculated. A rank correlation coefficient of *r* =.10, *r* =.30, and *r* =.50 indicates a small, moderate, and strong effect [59]. In hierarchical regression, parents’ worries were examined in relation to their child’s media use. Cohen’s *f* ^2^ was also calculated as an effect size. According to the guidelines by Cohen [59], *f* ² ≥ 0.02, *f* ² ≥ 0.15, and *f* ² ≥ 0.35 represent small, medium, and large effect sizes. The first model included the control variables of the child’s impairment, age, and gender. The second model included the variable of difficulties in restricting media consumption, and the third model included the parents’ perceptions of their child’s media preference and addiction. The fourth model adds the number of hours that children can cope without media without experiencing problems, and the final model adds the maximum daily media time that parents consider appropriate for their child. Prior to the hierarchical regression analysis, a dataset containing only parents’ worries and predictors was created, and all datasets with missing values were discarded to enable the analyses. The significance level was set at 5%.

## Results

There were no significant differences in terms of the number of digital devices available in the household (*U* = 3124.5, *z* = −0.88, *p-adj = *1) and the frequency of digital media use (*U* = 2795.5, *z* = −1.82, *p-adj* = 1) between the ASD group (number of digital devices (*n* = 117): *Md* = 7; frequency (*n* = 116): *M* = 1.58, *SD* = 0.43) and the TD group (number of digital devices (*n* = 58): *Md* = 7; frequency (*n* = 58): *M* = 1.47, *SD* = 0.49) (for more information on media use, see supporting information, S1-S10 Tables). The maximum amount of time that parents consider to be appropriate for media use per day differed significantly between the ASD and TD groups, both during the week (*U* = 4713.5, *z* = −4.99, *p-adj* < .001, *r* = .73) and during weekends including holidays (*U* = 4292, *z* = −4.18, *p-adj* < .001, *r* = .7). Parents of children with ASD considered 120.57 minutes (*SD* = 81.93, *n* = 115) during the week and 182.54 minutes (*SD* = 92.69, *n* = 112) on weekends to be the maximum appropriate amount of time. The parents of TD children considered 63.75 minutes (*SD* = 33.05; *n* = 56) during the week and 122.27 minutes (*SD* = 58.1; *n* = 55) on weekends to be the maximum appropriate duration. As the maximum time considered appropriate for children’s media use differed significantly between weekdays and weekends (**U* *= 7705.5, *z* = −7.4, *p-adj* < .001, *r* = .27), the mean of the two values was calculated for further analysis. Similarly, the mean maximum acceptable media use time differed significantly between the ASD and TD groups (*U* = 4475, *z* = −4.73, *p-adj* < .001, *r* = .73). On average, parents of children with ASD considered 150.76 minutes (*SD* = 81.23; *n* = 112; range = 30−390) appropriate, whereas parents of TD children considered 93.05 minutes (*SD* = 44; *n* = 55; range = 30–180) appropriate. There is also a significant difference in how often parents give their children digital devices to help self-regulate when they are unwell (*U* = 4571, *z* = −4.24, *p-adj* < .001, *r* = .69; ASD: *M* = 1.77, *SD* = 1.02, *n* = 116; TD: *M* = 1.05, *SD* = 0.89, *n* = 57). Overall, parents’ assessments of how long their children can cope without using media in everyday life differed between the two groups (*U* = 1884, *z* = −4.96, *p-adj* < .001, *r* = .28). Among the 117 parents of children with ASD, 51.28% (*n* = 60) reported that their child could manage a whole day or more without using media. Among the 58 parents of TD children, 81.04% (*n* = 47) said the same. For more information, see supporting information, S11-S12 Tables.

There was a significant difference in parents’ assessment of their children’s preference for and addiction to digital media in everyday life between the groups (*U* = 4893.5, *z* = −5.01, *p-adj* < .001, *r* = .73; ASD: *M* = 4.88, *SD* = 1.81, *n* = 115; TD: *M* = 3.45, *SD* = 1.50, *n* = 58). The two groups also differ in terms of the child’s and parent’s media skills (*U* = 4348, *z* = −3.37, *p-adj* = .013, *r* = .65; ASD: *M* = 5.87, *SD* = 1.71, *n* = 116; TD: *M* = 4.95, *SD* = 1.39, *n* = 57). Additionally, the two groups differed significantly in terms of parents’ assessments of restrictions and challenges in regulating media use (*U* = 5248.5, *z* = −6.42, *p-adj* < .001, *r* = .8; ASD: *M* = 4.01, *SD* = 2.17, *n* = 115; TD: *M* = 2.09, *SD* = 1.31, *n* = 57). Furthermore, there were statistically significant differences in parents’ concerns about media addiction between the two groups (*U* = 4743.5, *z* = −4.29, *p-adj* < .001, *r* = .7; ASD: *M* = 5.52, *SD* = 2.68, *n* = 117; TD: *M* = 3.64, *SD* = 2.64, *n* = 58). Concerns about their children losing their real-world experience through media use were not statistically significant (*U* = 4203, *z* = −2.8, *p-adj* = .086; ASD: *M* = 3.97, *SD* = 2.62, *n* = 115; TD: *M* = 2.93, *SD* = 2.45, *n* = 58). A significant difference was found in the concerns of parents in both groups regarding the negative effects of media use on health and behavior (*U* = 3683.5, *z* = −3.19, *p-adj* = .025, *r* = .65; ASD: *M* = 2.9, *SD* = 1.84, *n* = 99; TD: *M* = 2.15, *SD* = 1.74, *n* = 57). Overall, the concerns of parents with children with ASD differ significantly from those of parents with TD children (*U* = 3647, *z* = −3.3, *p-adj* = .016, *r* = .66; ASD: *M* = 3.49, *SD* = 1.76, *n* = 97; TD: *M* = 2.69, *SD* = 1.84, *n* = 57). However, parents’ concerns about their children’s media use are not affected by the child’s gender (*U* = 2659, *z* = −1.24, *p-adj* = 1; boys: *M* = 3.28, *SD* = 1.82, *n* = 112; girls: *M* = 2.95, *SD* = 1.83, *n* = 42). No differences were found between the two groups in terms of parents’ concerns in relation to media use and ASD symptoms (*U* = 3348, *z* = −2.04, *p-adj* = .697; ASD: *M* = 1.87, *SD* = 1.86, *n* = 101; TD: *M* = 1.44, *SD* = 1.51, *n* = 58).

Parents’ concerns about their child’s media addiction were significantly correlated with their perception of a preference for digital media and addiction (*r*_*s*_ = 0.73, *p-adj* < .001), the challenges of limiting media use (*r*_*s*_ = 0.61, *p-adj* < .001), and their assessment of how many hours their child can cope without media without experiencing problems (*r*_*s*_ = −0.53, *p-adj* < .001). Parents’ concerns about the negative effects of digital media on behavior and health were significantly correlated with their perceptions of a preference for and addiction to digital media (*r*_*s*_ = 0.58, *p-adj* < .001). The maximum daily screen time that parents consider appropriate correlates significantly with the problems of restricting media use (*r*_*s*_ = 0.42, *p-adj* < .001), their assessment of how many hours their child can cope without media without experiencing problems (*r*_*s*_ = −0.37, *p-adj* < .001), and the child’s age (*r*_*s*_ = 0.31, *p-adj* < .001). However, there was no significant correlation between parents’ concerns and their child’s age (*r*_*s*_ = 0.06, *p-adj* = 1) and the maximum daily screen time that parents considered appropriate (*r*_*s*_ = 0.18, *p-adj* = .372). A significant correlation was found between parents’ concerns and their assessment of how long the child can cope without digital media without experiencing problems (*r*_*s*_ = −0.55, *p-adj* < .001), parents’ perception of a preference for digital media and addiction (*r*_*s*_ = 0.68, *p-adj* < .001), and challenges limiting media use (*r*_*s*_ = 0.56, *p-adj* < .001).

The control variables of impairment, the child’s age and gender were used in a hierarchical regression analysis to examine the influence of various predictors on parents’ concerns regarding their child’s media use. The impairment variable was significant (*p* = .036). However, age and gender of the child had no significant influence (*p* > .05). The model incorporating the control variables was not significant (*F*(3,143) = 1.933, *p* = .127, *adjusted R²* = 0.02, *f* ^2^ = .02). Various predictors were stepwise added to further models. First, the variable ‘difficulties in limiting children’s media consumption’ was added. This model was significant (*F*(4,142) = 7.412, *p* <.001, *adjusted R²* = 0.15, *f* ^2^ =.18). Next, parents’ perceptions of media preferences and addiction were added. This model was also significant (*F*(5,141) = 18.94, *p* < .001, *adjusted R²* = 0.38, *f* ^2^ = .61). The next model also included a predictor of how many hours a child could cope without media before experiencing problems, and this predictor was significant (*F*(6,140) = 16.92, *p* < .001, *adjusted R²* = 0.4, *f* ^2^ = .67). Finally, the average amount of daily media time that parents perceived as appropriate was added to the final model, which was also significant (*F*(7,139) = 14.89, *p* < .001, *adjusted R²* = 0.4, *f* ^2^ = .67). The models were then compared with each other. Adding the predictor of difficulty in restricting media use to the model containing the control variables resulted in a significant increase in explained variance (*F*(1,142) = 32.53, *p* < .001). Similarly, adding parents’ perceptions of media preference and addiction, as well as how many hours children can easily cope without digital media, led to a significant increase in explained variance (*F*(1,141) = 55.71, *p* < .001 and *F*(1,140) = 4.49, *p* = .036). However, adding the maximum amount of time that parents considered appropriate to the final model had no significant effect on parents’ concerns about media use (*F*(1,139) = 1.99, *p* = .161). Overall, hierarchical regression revealed that difficulties with restricting media use, perceptions of media preference and addiction, and the amount of time children can easily cope without media are significant predictors of parental concern about their child’s media use. For the full results of any hierarchical regression analysis, see supporting information, S5 Appendix.

## Discussion

This study examined whether the attitudes and concerns of parents differ between those with and without children with ASD. Essentially, this study revealed no difference in the amount of time spent using digital media between the two groups, which is consistent with the results of Pliska et al. [14]. With respect to parents’ concerns about their child’s media use, significant predictors were the difficulty of limiting media time, parents’ perceptions that their child has a preference for and dependence on digital media, and the number of hours the child can cope without media. These predictors also correlate with parental concerns. The more parents worry about their child’s media use, the more problematic it is for them to restrict it. Conversely, the less parents worry, the easier it is for them to restrict their child’s media use. This result may therefore be related to findings by Kuo et al. [15], who showed that parents’ concerns about media use are linked to mediation strategies, such as restricting use. Parents who are more concerned would therefore be more likely to restrict their children’s media use, and thus more likely to encounter problems, than parents who are less concerned and therefore less likely to resort to restrictive control. Additionally, concerns increase with the perception that a child has a preference for and is dependent on digital media. On the other hand, the latter predictor correlates negatively with parents’ concerns. This means that parents who say that their child can manage without digital media for longer without problems are less worried. This suggests that, according to parents, TD children can manage without media for longer than children with ASD without parents experiencing any problems in them. This result is consistent with the findings of a study by Coyne et al. [35], which showed that frequent use of media for emotional regulation was associated with more extreme emotions when the media was removed. Furthermore, the frequent use of media for emotional regulation was also associated with more problematic media use [35], which is also indicated by the findings of this study. The amount of time that parents say their child can cope without media correlates with the maximum amount of media time they consider appropriate. Therefore, it is not surprising that parents of children with ASD consider more time spent on digital media to be appropriate than parents of TD children. This may be related to the fact that parents of children with ASD find it more difficult to restrict their child’s media consumption. There is also a significant correlation between the maximum appropriate time and the difficulty of restricting media use. As Pliska et al. [14] recently demonstrated, parents of children with ASD find it more challenging to limit their children’s media consumption than parents of TD children. This is important, given that a study by Konca [4] stated that limiting screen time is the most common strategy for regulating children’s digital technology use. However, parents’ concerns were not correlated with the maximum appropriate media time reported. This may also be because parents attribute more media skills to their child with ASD than to their TD child. Therefore, our finding that the maximum appropriate media time reported did not correlate with parental concerns and was not a predictor of them cannot support the findings of Laurie et al. [25], who reported a relationship between parental concerns about their children with ASD using technology and the amount of time spent using technology. In our study, however, we found that the more parents perceived their child to have a preference for digital media, the more they worried about media addiction and its negative effects on their child’s health and behavior. Overall, parents with a child with ASD are more concerned about media addiction than parents with TD children. This finding lends weight to the evidence of Yuan et al. [7] that individuals with ASD are at a greater risk of becoming addicted to screens than those without ASD. In general, concerns about media addiction may be related to the fact that parents of children with ASD being more likely to perceive their child as having a preference for, and being addicted to, digital media than parents of TD children. However, concerns that children are losing touch with the real world through media consumption do not differ between the two groups. This may also be related to the previously mentioned differences in restricting media use and the number of hours a child can cope without media, as these factors correlate with concerns about media addiction. Furthermore, parents of children with ASD were found to be more concerned about the negative effects of media use on their children’s health and behavior than parents of TD children, which supports the findings of MacMullin et al. [26]. However, concerns were not very high in either group.

No differences were observed between the groups regarding concerns about the development and intensification of ASD symptoms. Overall, concerns in this regard were stated as being very low, indicating that there is no concern that media use increases ASD symptoms. Notably, however, only concerns about the development and intensification of ASD symptoms were considered. However, it would also be worthwhile to ask whether media use improves ASD symptoms and, if so, whether they are absent or barely present during digital media use. For example, studies show that communication skills can be developed through digital media [33]. Additionally, parents of children with ASD state that they give their child digital media more often when they are unwell to help them self-regulate than do parents of TD children. This is consistent with findings by Gueron-Sela [34] and Coyne et al. [35] that parents use media to regulate their child’s distress and manage their child’s difficult emotions, respectively. However, the question arises as to whether parents are aware of this. It is possible that parents of children with ASD are more aware of it because screen media meet the sensory stimulation and solitary activity needs of children with ASD [27,28], and these parents are generally more engaged with their child’s media use than parents of TD children. On one hand, this would explain, the group difference regarding giving the child media to self-regulate and on the other hand the small sample size in the TD group.

In summary, the two groups of parents have different concerns, with those of children with ASD worrying more than those of children with TD. This lends weight to the assumption by Laurie et al. [25] that the common concerns of parents may be exacerbated if their child has ASD. However, it should be noted that the concerns in both groups are not particularly pronounced. This suggests that parents of children either with or without ASD are not particularly concerned about their child’s media use.

### Limitations

Overall, the results should be interpreted with caution. This is first because of the small sample size and second because it was not possible to calculate exact *p*-values for the correlations due to ties (i.e., equal values). Additionally, it is possible that only individuals with a personal interest in the topic participated in the survey. This could have distorted the results and may also explain why the TD group was so small, despite many attempts to reach more families with a TD child. Perhaps digital media have simply become part of everyday life, to the extent that parents unconsciously integrate them into their TD children’s daily routine without worrying about the issue of media use. In addition, no standardized questionnaire has been used, as, to the best of our knowledge, no standardized questionnaire is yet available for recording concerns related to media use. However, the values for internal consistency (reliability) are satisfactory, except for the media skills scale. Furthermore, comorbid impairments in the ASD group were not considered in the results. It is possible that comorbid impairment influenced the results. However, most of the children in the ASD group also had ADHD. As a study by Hill et al. [38] showed that screen time was comparable in the ASD and ADHD groups and that earlier screen use was associated with more severe symptoms of ASD and ADHD, it can be assumed that the comorbid disorder did not greatly influence our results. Another limitation is the age range of 6–11 years, as this study revealed a positive correlation between age and the maximum amount of time that parents deemed appropriate for media use. Therefore, this sample may include children who have been exposed to media for a long time and have more experience with digital media, and children who have not. However, this does not necessarily affect the validity of the study’s results, as age was not a significant factor in the hierarchical regression analysis and did not correlate with parental concerns. Furthermore, the study did not ask about parents’ media consumption. Nevertheless, this information is important, as a study by Matthes et al. [23] showed that excessive smartphone use is associated with a loss of control over children’s smartphone use. Parents’ media consumption can therefore influence their children’s media use and worries.

### Implications

Future studies should also survey and consider parents’ media consumption. The influence of parents’ media consumption on their children’s media use and their concerns in this regard should also be investigated. Additionally, the analyses should be repeated with a larger sample to create age- or media consumption-based clusters and examine parental concerns about media use. Overall, the study concluded that parental concerns about media use hardly differ between children with and without ASD and are generally very low. However, the study also revealed that parents with ASD find it more difficult to limit their child’s media consumption, stating less often that the child can cope well without media and are increasingly aware that their child is dependent on digital media. For this reason, parents with a child with ASD should particularly be supported in limiting their child’s media consumption if necessary. Despite this, it should also be emphasized that parents of children with ASD often rate their child’s media skills better and use digital devices as a regulatory strategy. In this context, a study should investigate whether ASD symptoms improve with media use and whether this could explain the greater recommended maximum media time. For example, Griffith [24] showed that parents’ more positive attitudes toward screen media were associated with higher media content consumption. Additionally, some studies suggest that the link between problematic screen use and ASD is likely because screen media fulfills the need for sensory stimulation and solitary activity [27,28] and helps individuals with ASD cope with social overstimulation [29]. A further study should examine the connections among high digital media consumption in children with ASD, the limited amount of time they can spend without digital media, parental difficulties in restricting their media use, and parents’ perceptions that digital media is beneficial for their children, helping them regulate themselves and develop social skills. Overall, a study should examine whether and to what extent children behave differently when using digital media and whether ASD symptoms may not be present. This is important to investigate in the context of digitalization and the development of digital screenings because digital screenings can work only if ASD symptoms appear in a digital setting.

## Conclusion

Overall, the study shows that parents of children with and without ASD are not particularly concerned about their children’s media use. However, parents of children with ASD are slightly more concerned about media addiction and its negative effects on their children’s health and behavior than parents of TD children. Predictors of parental concern about their child’s media use include difficulty in limiting use, perception of preference and addiction, and how long children can cope without media. Furthermore, no differences were found in the concerns of parents with children with and without ASD regarding the development or intensification of ASD symptoms, although hardly any concerns were expressed in this regard. It should be noted that the results are based on a small sample, and that the survey may have been completed primarily by parents who are already actively engaged with the issue of their children’s media use. Additionally, parental media use, which can influence views and concerns about children’s media use, was not considered. These aspects should be considered in a new study. Furthermore, the study only considered the development and intensification of ASD symptoms, and did not ask whether children’s behavior and ASD symptoms improved as a result of media consumption. A new study should examine the positive and negative effects of media use on the behavior of children with and without ASD.

## Supporting information

S1 Appendix Questionnaire in German. (PDF)

S2 Appendix Translated questionnaire in English. (PDF)

S3 Appendix Subscales (translated in English). (PDF)

S4 Appendix R code: Data analysis. (PDF)

S5 Appendix Output hierarchical regression analysis. (PDF)

S6 Appendix Study data. (XLSX)

S1 Table Availability of digital media in the home and frequency of use (children with ASD: *n* = 117, TD children: *n* = 58). (PDF)

S2 Table Own property of digital media (children with ASD: *n* = 117, TD children: *n* = 58). (PDF)

S3 Table Devices available and usable in the children’s room. (PDF)

S4 Table Average time spent on digital media per day. (PDF)

S5 Table The maximum amount of media time that parents consider appropriate per day (in min). (PDF)

S6 Table Age from which the child uses digital media almost daily. (PDF)

S7 Table Reasons for using digital media as named by parents across both groups (children with ASD: *n* = 117, TD children: *n* = 58). (PDF)

S8 Table Situations in which the child is given a digital device. (PDF)

S9 Table Parents’ awareness of their children’s use of digital media. (PDF)

S10 Table Rules for media use. (PDF)

S11 Table The amount of time that the child can cope without using media in their everyday life. (PDF)

S12 Table Necessity of media use for the child currently. (PDF)

S13 Table Code booklet for the Excel file containing the study data. (PDF)
