# Supplementary material for: Media use among children with ASD: Perspectives and concerns of parents
Source: PLoS One. 2025 Oct 13;20(10):e0332504. doi: 10.1371/journal.pone.0332504 (PMC12517494; doi:10.1371/journal.pone.0332504)
Supplement: S1 Appendix — (PDF) [file pone.0332504.s001.pdf]

# Fragebogen zur Mediennutzung

Liebe Erziehungsberechtigte,

die neuen Medien wie Smartphones oder Tablets gehören nicht nur zu unserem Alltag, auch unsere Kinder nutzen sie. Vielleicht haben auch Sie sich schon einmal Gedanken gemacht, womit sich Ihr Kind da genau beschäftigt. Vielleicht fragen Sie sich auch manchmal, ob die Medien Ihrem Kind guttun. Diese Fragen könnten Sie vielleicht besonders dann beschäftigen, wenn Sie sich Sorgen um die Entwicklung Ihres Kindes machen.

In einer Studie der TU Dortmund wenden wir uns an Erziehungsberechtigte von Kindern zwischen 6 und 11 Jahren, um Ihre Perspektive als Verantwortliche für das Kind besser kennen zu lernen. Wir haben hierzu diesen Fragebogen entwickelt, der etwa 15-20 Minuten Ihrer Zeit beansprucht.

Bitte antworten Sie die nachfolgenden Fragen ehrlich. Ihre Antworten sind anonym, sodass ein Rückschluss auf Ihr Kind oder Ihre Familie nicht möglich ist.

Sollten Sie mehrere Kinder im Grundschulalter haben, bitten wir Sie darum, jeweils einen Fragebogen pro Kind auszufüllen.

**Einverständnis**

Bitte lesen Sie die folgenden Punkte durch und bestätigen Sie Ihre Zustimmung:

- Ich verstehe, dass meine Teilnahme an dieser Studie freiwillig ist und dass ich das Recht habe, jederzeit ohne Konsequenzen abzubrechen.
- Ich verstehe, dass meine Antworten anonymisiert bleiben und nicht mit meiner Identität verknüpft werden.
- Ich bin damit einverstanden, dass die Informationen, die ich gebe, in Abschlussarbeiten, wissenschaftlichen Publikationen und Präsentationen, ohne identifizierende Details, veröffentlicht werden können.
- Ich verstehe, dass ich meine Einwilligung jederzeit widerrufen kann. Mir entstehen daraus keine negativen Folgen.
- Ich verstehe, dass bei Widerspruch der Einwilligung keine Löschung meiner Daten erfolgen kann, da meine Daten anonym erfasst werden.
- Ich habe die Hinweise zu Datennutzung und zum Datenschutz gelesen, verstanden und stimme diesen zu.

☐ Ich stimme zu

**Hinweise zur Datennutzung und zum Datenschutz**

Die nachfolgenden Hinweise entsprechen der aktuellen Datenschutzgrundverordnung.

- Alle erfassten Daten werden selbstverständlich vertraulich behandelt.
- Die Daten werden nur durch die angegebene beteiligte Person ausgewertet sowie durch Personen, die schriftlich die Einhaltung der datenschutzrechtlichen Bestimmungen für dieses Projekt unterschrieben haben. Im Rahmen der Auswertungen werden die Daten anonymisiert, d. h., es werden sämtliche Hinweise, die Rückschlüsse auf Personen ermöglichen würden, entfernt.
- Die Forschenden sind schriftlich zur Einhaltung der datenschutzrechtlichen Bestimmungen verpflichtet.
- Die gewonnenen Erkenntnisse werden ausschließlich zur Veröffentlichung in Publikationen und ggf. im Rahmen von Abschlussarbeiten und Konferenzen verwendet. In diesem Rahmen sind alle Daten anonymisiert und erlauben zu keiner Zeit Rückschlüsse auf Ihre Person.
- Es werden keine anderen Zwecke und Ziele verfolgt.
- Die Daten werden ausschließlich im genannten Kontext verwendet.
- Die Einwilligung zur Teilnahme und Zustimmung zur Verwendung der Daten ist freiwillig.
- Die Teilnehmenden haben das Recht auf eine umfangreiche Auskunftserteilung zu den zu Ihrer Person bezogenen gespeicherten Daten (personenbezogene Daten sind z.B. Alter, Bildungsabschluss, Beeinträchtigung).
- Durch eine Verweigerung der Einwilligung entstehen keine Nachteile. Die Einwilligung kann jederzeit mit Wirkung für die Zukunft widerrufen werden. Jedoch kann aufgrund der anonymisierten Erhebung der Daten kein Rückschluss auf den von Ihnen angegeben Angaben gezogen werden, um diese zu löschen.

- Wenn Sie der Auffassung sind, dass ich bei der Verarbeitung Ihrer Daten datenschutzrechtliche Vorschriften nicht beachtet haben, können Sie sich mit einer Beschwerde an den Datenschutzbeauftragten der TU Dortmund wenden, der Ihre Beschwerde prüfen wird: Die Kontaktdaten des Datenschutzbeauftragten lauten: TU Dortmund; Dr. Kai-Uwe Loser; Datenschutzbeauftragter; August-Schmidt-Str. 4; 44227 Dortmund; Telefon: 0231 755-2593.

**Digitale Medien**

Sollten Sie mehrere Kinder im Grundschulalter haben, bitten wir Sie darum, jeweils einen Fragebogen pro Kind auszufüllen.

1. Welche digitalen Medien sind in Ihrem Haushalt vorhanden? Wie oft nutzt Ihr Kind diese Geräte?

– Mehrfachnennungen möglich –

| Gerät | im Haushalt vorhanden | nutzt mein Kind | | | |
|---|---|---|---|---|---|
| | | nie | ein paar Mal im Monat | mehrmals pro Woche | täglich |
| PC / Laptop | ☐ | ☐ | ☐ | ☐ | ☐ |
| Tablet | ☐ | ☐ | ☐ | ☐ | ☐ |
| Smartphone | ☐ | ☐ | ☐ | ☐ | ☐ |
| Spielekonsole | ☐ | ☐ | ☐ | ☐ | ☐ |
| Fernseher | ☐ | ☐ | ☐ | ☐ | ☐ |
| Radio / Musikrecorder (inkl. Tonieboxen) | ☐ | ☐ | ☐ | ☐ | ☐ |
| Digitale Assistenten (Alexa, ...) | ☐ | ☐ | ☐ | ☐ | ☐ |
| SMART-Toys[1] (vernetzte Spielzeuge, z.B. tiptoi) | ☐ | ☐ | ☐ | ☐ | ☐ |
| Sonstiges: ________________ | ☐ | ☐ | ☐ | ☐ | ☐ |

[1] Vernetztes Spielzeug: Spielzeug mit Sensoren oder einer künstlichen Intelligenz; Aufbau einer Bluetooth- oder Internet-Verbindung bei Anwendung; Beispiel: Bücher oder Spielzeug mit begleitenden Apps oder auch Teddybären und Puppen mit Sprachausgabe

2. Besitzt Ihr Kind...

| | Ja | Nein |
|---|---|---|
| ... ein eigenes Smartphone? | ☐ | ☐ |
| ... einen eigenen Computer/ Laptop? | ☐ | ☐ |
| ... ein eigenes Tablet / iPad? | ☐ | ☐ |
| ... einen eigenen Fernseher? | ☐ | ☐ |
| ... eine eigene Spielekonsole? | ☐ | ☐ |

3. Sind Geräte wie Smartphone, Computer/PC, Tablet/iPad, Fernseher o.ä. im (eigenen oder geteilten) Kinderzimmer Ihres Kindes verfügbar und zur Nutzung erlaubt?

☐ Ja ☐ Nein ☐ Wir haben ausschließlich einen gemeinsamen Wohnraum

4. Wie viel Zeit verbringt Ihr Kind im Durchschnitt täglich mit den folgenden Medien?

| | An einem Tag in der Woche | | | | | An einem Tag am Wochenende/Feiertag/Ferien | | | | |
|---|---|---|---|---|---|---|---|---|---|---|
| | nie | bis zu 30 min | 30 min bis 2 Std. | 2 Std. bis 4 Std. | 4 Std. und mehr | nie | bis zu 30 min | 30 min bis 2 Std. | 2 Std. bis 4 Std. | 4 Std. und mehr |
| PC / Laptop | ☐ | ☐ | ☐ | ☐ | ☐ | ☐ | ☐ | ☐ | ☐ | ☐ |
| Tablet | ☐ | ☐ | ☐ | ☐ | ☐ | ☐ | ☐ | ☐ | ☐ | ☐ |
| Smartphone | ☐ | ☐ | ☐ | ☐ | ☐ | ☐ | ☐ | ☐ | ☐ | ☐ |
| Spielekonsole | ☐ | ☐ | ☐ | ☐ | ☐ | ☐ | ☐ | ☐ | ☐ | ☐ |
| Musikrecorder (inkl. Tonieboxen, Hörspiele) | ☐ | ☐ | ☐ | ☐ | ☐ | ☐ | ☐ | ☐ | ☐ | ☐ |
| Fernseher (inkl. Streaming-dienste wie Netflix, Disney+) | ☐ | ☐ | ☐ | ☐ | ☐ | ☐ | ☐ | ☐ | ☐ | ☐ |

5. Ab welchem Alter hat Ihr Kind die digitalen Medien (Smartphone, Tablet, etc.) fast täglich genutzt?

☐ mit ca. _________ Jahren

☐ mein Kind nutzt digitale Medien bisher nicht täglich

➔ Falls angekreuzt: Ab welchem Alter würden Sie Ihrem Kind erlauben digitale Medien fast täglich zu nutzen? mit ca. _________ Jahren

6. Wie viel Medienzeit pro Tag empfinden Sie für Ihr Kind als maximal angemessen?

| An einem Tag in der Woche | An einem Tag am Wochenende/Feiertag/Ferien |
|---|---|
| ________Stunden ______ Minuten | _______Stunden ______ Minuten |

7. Was macht Ihr Kind, wenn es digitale Medien nutzt?

| | nie | ein paar Mal im Monat | mehrmals pro Woche | täglich |
|---|---|---|---|---|
| Unterhaltungsspiele | ☐ | ☐ | ☐ | ☐ |
| Lernspiele | ☐ | ☐ | ☐ | ☐ |
| Fotos ansehen | ☐ | ☐ | ☐ | ☐ |
| Fotografieren | ☐ | ☐ | ☐ | ☐ |
| Musik/Hörspiele hören ( inkl. Toniebox) | ☐ | ☐ | ☐ | ☐ |
| Filme/Videos ansehen | ☐ | ☐ | ☐ | ☐ |
| Programmieren | ☐ | ☐ | ☐ | ☐ |
| Recherchieren | ☐ | ☐ | ☐ | ☐ |
| Mit anderen chatten/sich unterhalten | ☐ | ☐ | ☐ | ☐ |
| Sonstiges: ________________ | ☐ | ☐ | ☐ | ☐ |

8. Inwieweit bekommen Sie mit, wann und wie Ihr Kind digitale Medien nutzt?

| (fast) gar nicht | zum Teil | vollständig |
|---|---|---|
| ☐ | ☐ | ☐ |

9. In welchen Situationen überlassen Sie Ihrem Kind ein digitales Gerät (z.B. Tablet, Handy)?

| | nie | manchmal | häufig | regelmäßig |
|---|---|---|---|---|
| Um Wartezeit zu überbrücken | ☐ | ☐ | ☐ | ☐ |
| Wenn sich mein Kind langweilt | ☐ | ☐ | ☐ | ☐ |
| Zur Lernunterstützung | ☐ | ☐ | ☐ | ☐ |
| Um selbst Zeit für Anderes zu haben | ☐ | ☐ | ☐ | ☐ |
| Als gemeinsame Familienzeit | ☐ | ☐ | ☐ | ☐ |
| Wenn es meinem Kind nicht gut geht | ☐ | ☐ | ☐ | ☐ |
| Wenn die eigenen Belastungsgrenzen erreicht sind | ☐ | ☐ | ☐ | ☐ |
| Um ungestört Haushaltstätigkeiten nachzugehen | ☐ | ☐ | ☐ | ☐ |
| Zur Belohnung | ☐ | ☐ | ☐ | ☐ |
| Sonstige: ________________________ | ☐ | ☐ | ☐ | ☐ |

10. Gibt es für Ihr Kind Regeln für die Mediennutzung?

☐ Nein

☐ Ja, und zwar: ______________________________________________________________

______________________________________________________________________________

11. Welche positiven Aspekte sehen Sie bei der Mediennutzung Ihres Kindes?
– Mehrfachnennungen möglich –

☐ keine

☐ Kontakt zu Freunden/Freundinnen

☐ bildet sich weiter/ lernt dabei

☐ entspannt sich

☐ Sonstiges: ____________________________

12. Welche negativen Aspekte sehen Sie bei der Mediennutzung Ihres Kindes
– Mehrfachnennungen möglich –

☐ keine

☐ hat weniger soziale Kontakte

☐ vernachlässigt die Schule

☐ ist ständig unter Spannung/ wirkt „überdreht“

☐ ist verträumt/ nicht aufnahmefähig

☐ Sonstiges: ____________________________

13. Wie viele Stunden würde Ihr Kind ohne die Nutzung von Medien im Alltag gut aushalten?

☐ gar nicht ☐ 1-2 Stunden ☐ 3-4 Stunden

☐ 5-6 Stunden ☐ 7-12 Stunden ☐ einen ganzen Tag

☐ ohne Probleme mehr als einen Tag

14. Wie schätzen Sie die Notwendigkeit der Mediennutzung für Ihr Kind aktuell ein?

☐ Nicht notwendig, weil: ___________________________________

☐ Praktisch, aber verzichtbar, weil: ___________________________________

☐ Unverzichtbar, weil: ___________________________________________

☐ Sonstige: __________________

15. Im Folgenden geht es um den Umgang von Ihrem Kind mit digitalen Medien im Alltag. Bitte geben Sie für jede Aussage an, wie häufig diese auf Ihr Kind zutrifft.

| | Trifft nie zu 1 | 2 | 3 | 4 | 5 | 6 | 7 | 8 | 9 | Trifft (fast) immer zu 10 |
|---|---|---|---|---|---|---|---|---|---|---|
| Mein Kind freut sich darauf, (wieder) digitale Medien zu nutzen. | ☐ | ☐ | ☐ | ☐ | ☐ | ☐ | ☐ | ☐ | ☐ | ☐ |
| Mein Kind taucht so in die digitalen Medien ein, dass es alles um sich herum vergisst. | ☐ | ☐ | ☐ | ☐ | ☐ | ☐ | ☐ | ☐ | ☐ | ☐ |
| Mein Kind bevorzugt digitale Medien, statt Zeit mit anderen Personen zu verbringen, z.B. Eltern, Freunde. | ☐ | ☐ | ☐ | ☐ | ☐ | ☐ | ☐ | ☐ | ☐ | ☐ |
| Mein Kind verbringt zu viel Zeit mit digitalen Medien. | ☐ | ☐ | ☐ | ☐ | ☐ | ☐ | ☐ | ☐ | ☐ | ☐ |
| Mein Kind schläft zu wenig wegen der digitalen Medien. | ☐ | ☐ | ☐ | ☐ | ☐ | ☐ | ☐ | ☐ | ☐ | ☐ |
| Mein Kind vergisst etwas Wichtiges, weil es digitale Medien genutzt hat. | ☐ | ☐ | ☐ | ☐ | ☐ | ☐ | ☐ | ☐ | ☐ | ☐ |
| Mein Kind erledigt Aufgaben zu Hause hastig, damit es früher digitale Medien nutzen kann. | ☐ | ☐ | ☐ | ☐ | ☐ | ☐ | ☐ | ☐ | ☐ | ☐ |
| Andere Menschen (z.B. Freunde der Familie, Großeltern, Lehrkräfte) sagen meinem Kind, dass es digitale Medien seltener nutzen sollte. | ☐ | ☐ | ☐ | ☐ | ☐ | ☐ | ☐ | ☐ | ☐ | ☐ |
| Mein Kind fühlt sich schlecht, wenn es gerade keine digitalen Geräte nutzen darf. | ☐ | ☐ | ☐ | ☐ | ☐ | ☐ | ☐ | ☐ | ☐ | ☐ |
| Mein Kind ist sicher im Umgang mit digitalen Medien. | ☐ | ☐ | ☐ | ☐ | ☐ | ☐ | ☐ | ☐ | ☐ | ☐ |
| Meinem Kind fällt es schwer, die Nutzung von digitalen Medien zu beenden. | ☐ | ☐ | ☐ | ☐ | ☐ | ☐ | ☐ | ☐ | ☐ | ☐ |
| Mir sagen andere Menschen (z.B. anderes Elternteil, Freunde, Großeltern), dass mein Kind digitale Medien seltener nutzen sollte. | ☐ | ☐ | ☐ | ☐ | ☐ | ☐ | ☐ | ☐ | ☐ | ☐ |
| Mein Kind nutzt digitale Medien weiterhin, obwohl es eigentlich aufhören soll. | ☐ | ☐ | ☐ | ☐ | ☐ | ☐ | ☐ | ☐ | ☐ | ☐ |
| Die Medienzeit meines Kindes führt in der Familie zu Konflikten. | ☐ | ☐ | ☐ | ☐ | ☐ | ☐ | ☐ | ☐ | ☐ | ☐ |
| Mein Kind muss lange und/oder häufig digitale Geräte nutzen, um sich wieder gut oder entspannt zu fühlen. | ☐ | ☐ | ☐ | ☐ | ☐ | ☐ | ☐ | ☐ | ☐ | ☐ |
| Ich traue meinem Kind zu, dass es schon selbst entscheiden kann, in welcher Menge/Dauer es digitale Medien nutzt. | ☐ | ☐ | ☐ | ☐ | ☐ | ☐ | ☐ | ☐ | ☐ | ☐ |
| Das Verlangen meines Kindes nach digitalen Medien ist oft stärker ausgeprägt als ich es für gut halte. | ☐ | ☐ | ☐ | ☐ | ☐ | ☐ | ☐ | ☐ | ☐ | ☐ |
| Ich schränke die Mediennutzung meines Kindes ein. | ☐ | ☐ | ☐ | ☐ | ☐ | ☐ | ☐ | ☐ | ☐ | ☐ |
| Das Verlangen meines Kindes nach digitalen Medien ist so übermächtig, dass ich sie ihr/ihm dann doch erlaube. | ☐ | ☐ | ☐ | ☐ | ☐ | ☐ | ☐ | ☐ | ☐ | ☐ |
| Ich traue meinem Kind zu, dass es schon selbst entscheiden kann, welche digitalen Medien es nutzt. | ☐ | ☐ | ☐ | ☐ | ☐ | ☐ | ☐ | ☐ | ☐ | ☐ |
| Mir fällt es schwer, den Medienkonsum meines Kindes einzuschränken. | ☐ | ☐ | ☐ | ☐ | ☐ | ☐ | ☐ | ☐ | ☐ | ☐ |
| Ich vermeide negative Gefühle meines Kindes (z.B. Langeweile, Ärger, Trauer, Shutdowns) durch Mediennutzung. | ☐ | ☐ | ☐ | ☐ | ☐ | ☐ | ☐ | ☐ | ☐ | ☐ |
| Mir gelingt die Einschränkung der Mediennutzung meines Kindes. | ☐ | ☐ | ☐ | ☐ | ☐ | ☐ | ☐ | ☐ | ☐ | ☐ |
| Ich fühle mich sicher im Umgang mit digitalen Medien. | ☐ | ☐ | ☐ | ☐ | ☐ | ☐ | ☐ | ☐ | ☐ | ☐ |

| 16. Bitte beurteilen Sie, inwieweit Sie den Aussagen in Bezug zur Mediennutzung Ihres Kindes zustimmen. Hierbei geht es um Ihre eigenen Gedanken und Sorgen als Elternteil. | | | | | | | | | | |
|---|---|---|---|---|---|---|---|---|---|---|
| Aufgrund der Mediennutzung meines Kindes mache ich mir Sorgen, ... | Stimme nicht zu 1 | 2 | 3 | 4 | 5 | 6 | 7 | 8 | 9 | Stimme voll-kommen zu 10 |
| ... dass mein Kind zu viel Zeit mit digitalen Medien verbringt. | ☐ | ☐ | ☐ | ☐ | ☐ | ☐ | ☐ | ☐ | ☐ | ☐ |
| ... dass mein Kind die realen Freunde verliert. | ☐ | ☐ | ☐ | ☐ | ☐ | ☐ | ☐ | ☐ | ☐ | ☐ |
| ... dass mein Kind sich körperlich gehen lässt. | ☐ | ☐ | ☐ | ☐ | ☐ | ☐ | ☐ | ☐ | ☐ | ☐ |
| ... dass mein Kind mehr Zeit mit digitalen Geräten verbringt, als andere Kinder in dem Alter. | ☐ | ☐ | ☐ | ☐ | ☐ | ☐ | ☐ | ☐ | ☐ | ☐ |
| ... dass mein Kind aggressive Verhaltensweisen entwickelt. | ☐ | ☐ | ☐ | ☐ | ☐ | ☐ | ☐ | ☐ | ☐ | ☐ |
| ... dass mein Kind online gemobbt wird. | ☐ | ☐ | ☐ | ☐ | ☐ | ☐ | ☐ | ☐ | ☐ | ☐ |
| ... dass die Augen meines Kindes schlechter werden. | ☐ | ☐ | ☐ | ☐ | ☐ | ☐ | ☐ | ☐ | ☐ | ☐ |
| ... dass mein Kind den Bezug zur realen Welt verliert. | ☐ | ☐ | ☐ | ☐ | ☐ | ☐ | ☐ | ☐ | ☐ | ☐ |
| ... dass mein Kind süchtig wird. | ☐ | ☐ | ☐ | ☐ | ☐ | ☐ | ☐ | ☐ | ☐ | ☐ |
| ... dass mein Kind online an die falschen Leute gerät. | ☐ | ☐ | ☐ | ☐ | ☐ | ☐ | ☐ | ☐ | ☐ | ☐ |
| ... dass mein Kind eine Beeinträchtigung entwickelt. | ☐ | ☐ | ☐ | ☐ | ☐ | ☐ | ☐ | ☐ | ☐ | ☐ |
| ... dass die soziale Kompetenz meines Kindes abnimmt. | ☐ | ☐ | ☐ | ☐ | ☐ | ☐ | ☐ | ☐ | ☐ | ☐ |
| ... dass mein Kind online zu viel von sich preisgibt. | ☐ | ☐ | ☐ | ☐ | ☐ | ☐ | ☐ | ☐ | ☐ | ☐ |
| ... dass mein Kind das Interesse an nicht-medialen Tätigkeiten verliert. | ☐ | ☐ | ☐ | ☐ | ☐ | ☐ | ☐ | ☐ | ☐ | ☐ |
| ... dass ich mich nicht genug mit digitalen Medien auskenne. | ☐ | ☐ | ☐ | ☐ | ☐ | ☐ | ☐ | ☐ | ☐ | ☐ |
| ... dass mein Kind autistische Verhaltensweisen entwickelt. | ☐ | ☐ | ☐ | ☐ | ☐ | ☐ | ☐ | ☐ | ☐ | ☐ |
| ... dass sich mein Kind in der digitalen Welt verliert. | ☐ | ☐ | ☐ | ☐ | ☐ | ☐ | ☐ | ☐ | ☐ | ☐ |
| ... dass ich keine Kontrolle mehr über das Leben meines Kindes habe. | ☐ | ☐ | ☐ | ☐ | ☐ | ☐ | ☐ | ☐ | ☐ | ☐ |
| ... dass ich meinem Kind zu viel im Umgang mit digitalen Medien verbiete. | ☐ | ☐ | ☐ | ☐ | ☐ | ☐ | ☐ | ☐ | ☐ | ☐ |
| ... da ich keine Unterstützung in der Nutzung von digitalen Medien erhalte. | ☐ | ☐ | ☐ | ☐ | ☐ | ☐ | ☐ | ☐ | ☐ | ☐ |
| ... dass ich den Kontakt zu meinem Kind verliere. | ☐ | ☐ | ☐ | ☐ | ☐ | ☐ | ☐ | ☐ | ☐ | ☐ |
| ... dass mein Kind Autismus bekommt / bekommen hat. | ☐ | ☐ | ☐ | ☐ | ☐ | ☐ | ☐ | ☐ | ☐ | ☐ |
| ... dass ich meinem Kind zu viel im Umgang mit digitalen Medien zutraue. | ☐ | ☐ | ☐ | ☐ | ☐ | ☐ | ☐ | ☐ | ☐ | ☐ |
| ... da ich keine Unterstützung bei der Medienerziehung meines Kindes erhalte. | ☐ | ☐ | ☐ | ☐ | ☐ | ☐ | ☐ | ☐ | ☐ | ☐ |
| ... dass ich meinem Kind zu viele Freiheiten gebe. | ☐ | ☐ | ☐ | ☐ | ☐ | ☐ | ☐ | ☐ | ☐ | ☐ |

**Kind** (wenn Sie mehrere Kinder im Grundschulalter haben, beziehen Sie sich auf eins)

1. Wie alt ist Ihr Kind? ________Jahre ________ Monate

2. Welches Geschlecht hat Ihr Kind?
   ☐ weiblich ☐ männlich ☐ divers

3. Hat Ihr Kind eine diagnostizierte Beeinträchtigung?
   ☐ Nein ☐ Ja

***Falls nein:***
Liegt bei Ihrem Kind aktuell der Verdacht auf eine Beeinträchtigung vor?
☐ Nein
☐ Ja: (Mehrfachnennung)
- ☐ Autismus-Spektrum-Störung (ASS)
- ☐ Aufmerksamkeits-(Hyper)Aktivitätsstörung (AD(H)S)
- ☐ Sprachentwicklungsstörung
- ☐ Lese-Rechtschreibschwäche
- ☐ Rechenschwäche
- ☐ Lernstörung
- ☐ Sonstige: ____________________

***Falls ja:***
(Mehrfachnennung)
☐ Autismus-Spektrum-Störung (ASS)
☐ Aufmerksamkeits-(Hyper)Aktivitätsstörung (AD(H)S)
☐ Sprachentwicklungsstörung
☐ Lese-Rechtschreibschwäche
☐ Rechenschwäche
☐ Lernstörung
☐ geistige Behinderung
☐ Sonstige: ______________________________

4. Mein Kind kann sich sprachlich altersgemäß mit anderen verständigen.
   ☐ Trifft zu
   ☐ Trifft eingeschränkt zu
   ☐ Trifft nicht zu (keine verbale Sprache)

5. Hat Ihr Kind Geschwister?
   ☐ Nein
   ☐ Ja: Wie viele? ______ → _______ davon jünger; _______ davon älter

6. Welche Einrichtung besucht Ihr Kind zur Zeit?
   ☐ Kindergarten ☐ inklusiver Kindergarten
   ☐ Regelgrundschule ☐ inklusive Grundschule ☐ Förderschule
   ☐ Weiterführende Schule
   ☐ Sonstige: ___________________________

7. In welcher Klasse ist Ihr Kind? ___________ *(nur bei Schule)*

**Erziehungsberechtigte Person**

1. Ich bin ☐ Mutter ☐ Vater ☐ Sonstige: ______________________

2. Wie alt sind Sie?

   Ich bin _______ Jahre alt.

3. Haben Sie oder eine andere erziehungsberechtigte Person eine diagnostizierte Beeinträchtigung?
   ☐ Nein
   ☐ Ja: ______________________________________________
   ☐ Keine Angabe

4. Höchster Bildungsabschluss der erziehungsberechtigten Person(en)
   Erziehungsberechtigte Person 1 (z.B. Mutter):
   ☐ ohne Schulabschluss
   ☐ Hauptschulabschluss
   ☐ Realschulabschluss
   ☐ Fachhochschulreife („Fachabitur“)
   ☐ Allgemeine Hochschulreife („Abitur)
   ☐ (Fach-)Hochschulabschluss
   ☐ Promotion
   Erziehungsberechtigte Person 2 (z.B. Vater):
   ☐ ohne Schulabschluss
   ☐ Hauptschulabschluss
   ☐ Realschulabschluss
   ☐ Fachhochschulreife („Fachabitur“)
   ☐ Allgemeine Hochschulreife („Abitur)
   ☐ (Fach-)Hochschulabschluss
   ☐ Promotion

Sonstige Angaben, die im Rahmen dieser Studie von Interesse sein könnten:

________________________________________________________________________

________________________________________________________________________

________________________________________________________________________

________________________________________________________________________

*Herzlichen Dank für Ihre Teilnahme!*

Möchten Sie den Fragebogen noch für ein weiteres Kind ausfüllen? Dann klicken Sie bitte auf diesen Link
