# Supplementary material for: Media use among children with ASD: Perspectives and concerns of parents
Source: PLoS One. 2025 Oct 13;20(10):e0332504. doi: 10.1371/journal.pone.0332504 (PMC12517494; doi:10.1371/journal.pone.0332504)
Supplement: S2 Appendix — (PDF) [file pone.0332504.s002.pdf]

Questionnaire on media use

Dear parents and guardians,

New media such as smartphones and tablets are not only part of our everyday lives, our children also use them. Perhaps you have also wondered what exactly your child is doing with them. Perhaps you sometimes wonder whether the media is good for your child. These questions may be of particular concern to you if you are worried about your child's development.

In a study conducted by TU Dortmund University, we asked parents and guardians of children between the ages of 6 and 11 to get to know your perspective as the person responsible for the child. We have developed this questionnaire for this purpose, which will take about 15-20 minutes of your time.

Please answer the following questions honestly. Your answers are anonymous, so it is not possible to draw conclusions about your child or your family.

If you have several children of primary school age, please complete one questionnaire for each child.

Consent

Please read the following points and confirm your consent:

- I understand that my participation in this study is voluntary and that I have the right to withdraw at any time without consequence.
- I understand that my responses will remain anonymous and will not be linked to my identity.
- I agree that the information I provide may be published in theses, scientific publications and presentations without identifying details.
- I understand that I can withdraw my consent at any time. I will not suffer any negative consequences as a result.
- I understand that if I withdraw my consent, my data cannot be deleted as my data is collected anonymously.
- I have read, understood and agree to the information on data use and data protection.

☐ I agree to

Information on data use and data protection

The following information complies with the current General Data Protection Regulation.

- All data collected will of course be treated confidentially.
- The data will only be evaluated by the person involved and by persons who have signed a written agreement to comply with the data protection regulations for this project. The data will be anonymized during evaluation, i.e. all references that would allow conclusions to be drawn about individuals will be removed.
- The researchers are obliged in writing to comply with the data protection regulations.
- The findings obtained are used exclusively for publication in publications and, if applicable, in the context of theses and conferences. In this context, all data is anonymized and does not allow any conclusions to be drawn about your person at any time.
- No other purposes or objectives are pursued.
- The data will only be used in the stated context.
- Consent to participate and consent to the use of the data is voluntary.
- Participants have the right to obtain comprehensive information about the data stored about them (personal data includes, for example, age, educational qualifications, disability).
- Refusal to give consent will not result in any disadvantages. Consent can be withdrawn at any time with effect for the future. However, due to the anonymized collection of the data, no conclusions can be drawn about the information you have provided in order to delete it.

- If you are of the opinion that I have not complied with data protection regulations when processing your data, you can lodge a complaint with the Data Protection Officer at TU Dortmund University, who will examine your complaint: The contact details of the Data Protection Officer are: TU Dortmund University; Dr. Kai-Uwe Loser; Data Protection Officer; August-Schmidt-Str. 4; 44227 Dortmund; Telephone: 0231 755-2593.

Digital media

If you have several children of primary school age, please complete one questionnaire per child.

1. What digital media do you have in your household? How often does your child use these devices?

- Multiple answers possible -

Device	present in the household	Does my child			
		never	a few times a month	several times a week	daily
PC / laptop	☐	☐	☐	☐	☐
tablet	☐	☐	☐	☐	☐
Smartphone	☐	☐	☐	☐	☐
Games console	☐	☐	☐	☐	☐
Television	☐	☐	☐	☐	☐
Radio / music recorder (incl. Tonieboxes)	☐	☐	☐	☐	☐
Digital assistants (Alexa, ...)	☐	☐	☐	☐	☐
SMART toys[(1)] (networked toys, e.g. tiptoi)	☐	☐	☐	☐	☐
Other: ________________	☐	☐	☐	☐	☐

[1]Connected toys: toys with sensors or artificial intelligence; establishment of a Bluetooth or Internet connection when used; example: books or toys with accompanying apps or teddy bears and dolls with voice output

2. Does your child own...	Yes	No
... their own smartphone?	☐	☐
... their own computer/laptop?	☐	☐
... your own tablet / iPad?	☐	☐
... your own television?	☐	☐
... your own games console?	☐	☐

3. Are devices such as a smartphone, computer/PC, tablet/iPad, TV or similar available and permitted for use in your child's (own or shared) room?

   ☐ Yes ☐ No ☐ We only have a shared living space

4. On average, how much time does your child spend using the following media each day?

	One day a week					One day at the weekend/holiday/vacation				
	Never	up to 30 min	30 min to 2 hours	2 hours to 4 hours	4 hours and more	never	up to 30 min	30 min to 2 hrs	2 hours to 4 hours	4 hours and more
PC / laptop	☐	☐	☐	☐	☐	☐	☐	☐	☐	☐
tablet	☐	☐	☐	☐	☐	☐	☐	☐	☐	☐
Smartphone	☐	☐	☐	☐	☐	☐	☐	☐	☐	☐
Games console	☐	☐	☐	☐	☐	☐	☐	☐	☐	☐
Music recorder (incl. Tonieboxes, radio plays)	☐	☐	☐	☐	☐	☐	☐	☐	☐	☐
Television (incl. streaming services such as Netflix, Disney)+	☐	☐	☐	☐	☐	☐	☐	☐	☐	☐

5. At what age did your child start using digital media (smartphone, tablet, etc.) almost daily?

☐ at around _________ years old

☐ My child does not yet use digital media on a daily basis

➔ If ticked: From what age would you allow your child to use digital media almost daily? at approx. _________ years old

6. How much media time per day do you think is the maximum appropriate for your child?

One day a week	One day at the weekend/holiday/vacation
_______hours _____ minutes	______hours _____ minutes

7. What does your child do when using digital media?

	Never	a few times a month	several times a week	daily
Entertainment games	☐	☐	☐	☐
Educational games	☐	☐	☐	☐
View photos	☐	☐	☐	☐
Take photos	☐	☐	☐	☐
Listen to music/radio plays (incl. Toniebox)	☐	☐	☐	☐
Watching movies/videos	☐	☐	☐	☐
Programming	☐	☐	☐	☐
Researching	☐	☐	☐	☐
Chatting/conversing with others	☐	☐	☐	☐
Other: _______________	☐	☐	☐	☐

8. To what extent are you aware of when and how hoy your child uses digital media?

(almost) not at all	partly	completely
☐	☐	☐

9. In which situations do you let your child use a digital device (e.g. tablet, smartphone)?

	never	sometimes	often	regularly
To bridge waiting time	☐	☐	☐	☐
When my child is bored	☐	☐	☐	☐
To support learning	☐	☐	☐	☐
To have time for other things myself	☐	☐	☐	☐
As family time together	☐	☐	☐	☐
When my child is not feeling well	☐	☐	☐	☐
When my own stress limits are reached	☐	☐	☐	☐
To do household chores undisturbed undisturbed	☐	☐	☐	☐
As a reward	☐	☐	☐	☐
Other: ______________________	☐	☐	☐	☐

10. Does your child have rules for media use?

☐ No

☐ Yes, namely: ____

____

11. What positive aspects do you see in your child's media use?
- Multiple answers possible -

☐ None

☐ Contact with friends

☐ educates himself/herself/learns

☐ relaxes

☐ Sonstiges: __________________________

12. What negative aspects do you see in your child's use of media
- Multiple answers possible -

☐ none

☐ Has fewer social contacts

☐ neglects school

☐ is constantly under tension/ seems "over-excited"

☐ Is dreamy/ not receptive

☐ Sonstiges: __________________________

13. How many hours would your child be able to cope without using media in everyday life?

☐ Not at all ☐ 1-2 hours ☐ 3-4 hours

☐ 5-6 hours ☐ 7-12 hours ☐ a whole day

☐ without problems more than one day

14. How do you currently rate the necessity of media use for your child?

☐ Not necessary because: ________________________________

☐ Practical, but dispensable because: _________________________________

☐ Essential because: ____

☐ Other: _________________

15. The following is about your child's use of digital media in everyday life. For each statement, please indicate how often it applies to your child.										
	Never applies 1	2	3	4	5	6	7	8	9	(Almost) always true 10
My child is looking forward to using digital media (again).	☐	☐	☐	☐	☐	☐	☐	☐	☐	☐
My child is so immersed in digital media that he/she forgets everything around him/her.	☐	☐	☐	☐	☐	☐	☐	☐	☐	☐
My child prefers digital media to spending time with other people, e.g. parents, friends.	☐	☐	☐	☐	☐	☐	☐	☐	☐	☐
My child spends too much time with digital media.	☐	☐	☐	☐	☐	☐	☐	☐	☐	☐
My child sleeps too little because of digital media.	☐	☐	☐	☐	☐	☐	☐	☐	☐	☐
My child forgets something important because he/she has used digital media.	☐	☐	☐	☐	☐	☐	☐	☐	☐	☐
My child completes tasks at home in a hurry so that he/she can use digital media earlier.	☐	☐	☐	☐	☐	☐	☐	☐	☐	☐
Other people (e.g. family friends, grandparents, teachers) tell <u>my child</u> that he/she should use digital media less often.	☐	☐	☐	☐	☐	☐	☐	☐	☐	☐
My child feels bad when he/she is not allowed to use digital devices.	☐	☐	☐	☐	☐	☐	☐	☐	☐	☐
My child is confident in using digital media.	☐	☐	☐	☐	☐	☐	☐	☐	☐	☐
My child finds it difficult to stop using digital media.	☐	☐	☐	☐	☐	☐	☐	☐	☐	☐
Other people (e.g. other parent, friends, grandparents) tell <u>me</u> that my child should use digital media less often.	☐	☐	☐	☐	☐	☐	☐	☐	☐	☐
My child continues to use digital media even though he/she should stop.	☐	☐	☐	☐	☐	☐	☐	☐	☐	☐
My child's media time leads to conflicts in the family.	☐	☐	☐	☐	☐	☐	☐	☐	☐	☐
My child has to use digital devices for a long time and/or frequently to feel good or relaxed again.	☐	☐	☐	☐	☐	☐	☐	☐	☐	☐
I trust my child to be able to decide for himself/herself how much or how long he/she use digital media.	☐	☐	☐	☐	☐	☐	☐	☐	☐	☐
My child's desire for digital media is often more pronounced than I think is good.	☐	☐	☐	☐	☐	☐	☐	☐	☐	☐
I limit my child's media use.	☐	☐	☐	☐	☐	☐	☐	☐	☐	☐
My child's desire for digital media is so overwhelming that I allow him/her to use it after all.	☐	☐	☐	☐	☐	☐	☐	☐	☐	☐
I trust my child to be able to decide for himself/herself which digital media he/she use.	☐	☐	☐	☐	☐	☐	☐	☐	☐	☐
I find it difficult to limit my child's media consumption.	☐	☐	☐	☐	☐	☐	☐	☐	☐	☐
I avoid negative feelings in my child (e.g. boredom, anger, sadness, shutdowns) through media use.	☐	☐	☐	☐	☐	☐	☐	☐	☐	☐
I am successful in limiting my child's media use.	☐	☐	☐	☐	☐	☐	☐	☐	☐	☐
I feel confident in dealing with digital media.	☐	☐	☐	☐	☐	☐	☐	☐	☐	☐

16. Please rate the extent to which you agree with the statements relating to your child's media use. This is about your own thoughts and concerns as a parent.										
I am worried about my child's media use, ...	Disagree<br>1	2	3	4	5	6	7	8	9	Totally agree<br>10
... that my child spends too much time with digital media.	☐	☐	☐	☐	☐	☐	☐	☐	☐	☐
... that my child is losing real-life friends.	☐	☐	☐	☐	☐	☐	☐	☐	☐	☐
... that my child lets himself/herself go physically.	☐	☐	☐	☐	☐	☐	☐	☐	☐	☐
... that my child spends more time with digital devices than other children their age.	☐	☐	☐	☐	☐	☐	☐	☐	☐	☐
... that my child is developing aggressive behavior.	☐	☐	☐	☐	☐	☐	☐	☐	☐	☐
... that my child is being bullied online.	☐	☐	☐	☐	☐	☐	☐	☐	☐	☐
... that my child's eyes are getting worse.	☐	☐	☐	☐	☐	☐	☐	☐	☐	☐
... that my child is losing touch with the real world.	☐	☐	☐	☐	☐	☐	☐	☐	☐	☐
... that my child is becoming addicted.	☐	☐	☐	☐	☐	☐	☐	☐	☐	☐
... that my child gets involved with the wrong people online.	☐	☐	☐	☐	☐	☐	☐	☐	☐	☐
... that my child develops an impairment.	☐	☐	☐	☐	☐	☐	☐	☐	☐	☐
... that my child's social skills are declining.	☐	☐	☐	☐	☐	☐	☐	☐	☐	☐
... that my child reveals too much about himself/herself online.	☐	☐	☐	☐	☐	☐	☐	☐	☐	☐
... that my child is losing interest in non-media activities.	☐	☐	☐	☐	☐	☐	☐	☐	☐	☐
... that I am not familiar enough with digital media.	☐	☐	☐	☐	☐	☐	☐	☐	☐	☐
... that my child is developing autistic behavior.	☐	☐	☐	☐	☐	☐	☐	☐	☐	☐
... that my child is getting lost in the digital world.	☐	☐	☐	☐	☐	☐	☐	☐	☐	☐
... that I no longer have any control over my child's life.	☐	☐	☐	☐	☐	☐	☐	☐	☐	☐
... that I forbid my child to use digital media too much.	☐	☐	☐	☐	☐	☐	☐	☐	☐	☐
... because I don't receive any support in the use of digital media.	☐	☐	☐	☐	☐	☐	☐	☐	☐	☐
... that I am losing contact with my child.	☐	☐	☐	☐	☐	☐	☐	☐	☐	☐
... that my child is developing / has developed autism.	☐	☐	☐	☐	☐	☐	☐	☐	☐	☐
... that I am overconfident in my child's use of digital media.	☐	☐	☐	☐	☐	☐	☐	☐	☐	☐
... that I am not receiving any support with my child's media education.	☐	☐	☐	☐	☐	☐	☐	☐	☐	☐
... that I give my child too much freedom.	☐	☐	☐	☐	☐	☐	☐	☐	☐	☐

Child (if you have several children of primary school age, please refer to one)

1. How old is your child? ________years ________ months
2. What gender is your child?
   ☐ female☐ male☐ diverse
3. Does your child have a diagnosed impairment?
   ☐ No ☐ Yes

If no:
Is your child currently suspected of having an impairment?
☐ No
☐ Yes: (multiple answers)
- ☐ Autism spectrum disorder (ASD)
- ☐ Attention (hyper)activity disorder (AD(H)D)
- ☐ Language development disorder
- ☐ Reading and spelling disorder
- ☐ dyscalculia
- ☐ Learning disorder
- ☐ Other: ___________________

If yes:
(multiple choice)
- ☐ Autism spectrum disorder (ASD)
- ☐ Attention (hyper)activity disorder (AD(H)D)
- ☐ Language development disorder
- ☐ Reading and spelling disorder
- ☐ dyscalculia
- ☐ learning disorder
- ☐ intellectual disability
- ☐ Other: ___________________________

4. My child can communicate with others in an age-appropriate way.
   ☐ Applies
   ☐ Applies to a limited extent
   ☐ Does not apply (no verbal language)
5. Does your child have siblings?
   ☐ No
   ☐ Yes: How many? _____ → ______ of which younger; ______ of which older
6. Which institution does your child currently attend?
   ☐ Kindergarten☐ inclusive kindergarten
   ☐ Regular elementary school☐ Inclusive elementary school☐ Special school
   ☐ Secondary school
   ☐ Other: ________________________
7. Which class is your child in? __________ *(only for school)*

Person with parental authority

1. I am☐ Mother☐ Father☐ Other: ______________________

2. How old are you?

   I am ______ years old.

3. Do you or another legal guardian have a diagnosed impairment?
   ☐ No
   ☐ Yes: ____
   ☐ No answer

4. Highest educational qualification of the legal guardian(s)
   Legal guardian person 1 (e.g. mother):
   - ☐ without high school diploma
   - ☐ General Certificate of Education
   - ☐ intermediate-secondary school-leaving certificate
   - ☐ subject-related entrance qualification
   - ☐ higher education entrance qualification (A-levels)
   - ☐ University degree
   - ☐ Doctorate

   Legal guardian person 2 (e.g. father):
   - ☐ without high school diploma
   - ☐ General Certificate of Education
   - ☐ intermediate-secondary school-leaving certificate
   - ☐ subject-related entrance qualification
   - ☐ higher education entrance qualification (A-levels)
   - ☐ University degree
   - ☐ Doctorate

Other information that may be of interest in the context of this study:

____

____

____

____

Thank you very much for your participation!

Would you like to complete the questionnaire for another child? Then please click on this link
