# Supplementary material for: Media use among children with ASD: Perspectives and concerns of parents
Source: PLoS One. 2025 Oct 13;20(10):e0332504. doi: 10.1371/journal.pone.0332504 (PMC12517494; doi:10.1371/journal.pone.0332504)
Supplement: S3 Appendix — (PDF) [file pone.0332504.s003.pdf]

S3 Appendix. Subscales (translated in English)

The child's use of digital media in everyday life

Preference for digital media and media addiction (15 items, α = .9)

- My child is looking forward to using digital media (again).
- My child is so immersed in digital media that he/she forgets everything around him/her.
- My child prefers digital media to spending time with other people, e.g. parents, friends.
- My child spends too much time with digital media.
- My child doesn't sleep enough because of digital media.
- My child forgets something important because he/she have used digital media.
- My child completes task at home hastily so that he/she can use digital media earlier.
- Other people (e.g. family friends, grandparents, teachers) tell my child that he/she should use digital media less often.
- My child feels bad when they are not allowed to use digital devices.
- My child finds it difficult to stop using digital media.
- Other people (e.g. other parent, friend, grandparents) tell me that my child should use digital media less often.
- My child continues to use digital media even though he/she is supposed to stop.
- My child has to use digital devices for a long time andor frequently in order to feel good or relaxed again.
- My child's desire for digital media is often more pronounced than I think is good.
- My child's media time leads to conflicts in the the family.

Media skills (4 items, α = .56)

- My child is confident in using digital media.
- I trust my child to be able to decide for himself/herself how much/how long he/she use digital media.
- I trust my child to be able to decide for himself/herself which digital media he/she use.
- I feel confident in dealing with digital media.

Restrictions and challenges in regulating media use (5 items, α = .82)

- I limit my child's media use. *(recoded)*
- I find it difficult to limit my child's media consumption.
- I am able to limit my child's media use. *(recoded)*
- My child's desire for digital media is so overwhelming that I allow him/her to use it after all.
- I avoid negative feeling of my child (e.g. boredom, anger, sagness, shutdowns) through media use.

Parents' concerns about their children's media use

Media addiction (3 items, α = .82)

- I am worried about my child's media use, that my child spend too much time with digital media.
- I am worried about my child's media use, that my child is becoming addicted.
- I am worried about my child's media use, that my child spends more time with digital devices than other children their age.

Loss of connection to the real world (4 items, α = .89)

- I am worried about my child's media use, that that my child is losing real-life friends.
- I am worried about my child's media use, that my child is losing touch with real world.
- I am worried about my child's media use, that my child is getting lost in the digital world.
- I am worried about my child's media use, that my child is losing interest in non-media activities.

Negative effects on health and behavior (7 items, α = .85)

- I am worried about my child's media use, that my child is letting himself/herself go physically.
- I am worried about my child's media use, that my child is developing aggressive behavior.
- I am worried about my child's media use, that my child's eyes are getting worse.
- I am worried about my child's media use, that my child will develop an inpairment.
- I am worried about my child's media use, that my child's social skills are declining.
- I am worried about my child's media use, that my child is developing autistic behavior.
- I am worried about my child's media use, that my child is developing/has developed autism.

Worries about online dangers, loss of control, and parental media skills (8 items, α = .84)

- I am worried about my child's media use, that I am not familiar enough with digital media.
- I am worried about my child's media use, that I no longer have any control over my child's life.
- I am worried about my child's media use, that I forbid my child to use digital media too much.
- I am worried about my child's media use, that I am losing contact with my child.
- I am worried about my child's media use, that I am overconfident in my child's use of digital media.
- I am worried about my child's media use, that I give my child too much freedom.
- I am worried about my child's media use, that my child is being bullied online.
- I am worried about my child's media use, that my child gets involved with the wrong people online.

No support as a reason for worries (2 items, α = .78)

- I am worried about my child's media use, because I don't receive any support in the use of digital media.
- I am worried about my child's media use, because I don't receive any support with my child's media education.

Concerns about media use and ASD symptoms (2 items, α = .67)

- I am worried about my child's media use, that my child is developing autistic behavior.
- I am worried about my child's media use, that my child is developing/has developed autism.
