# Supplementary material for: Media use among children with ASD: Perspectives and concerns of parents
Source: PLoS One. 2025 Oct 13;20(10):e0332504. doi: 10.1371/journal.pone.0332504 (PMC12517494; doi:10.1371/journal.pone.0332504)
Supplement: S4 Appendix — (PDF) [file pone.0332504.s004.pdf]

```
getwd()
library(openxlsx2)
dat <- read_xlsx("survey_data_final.xlsx")
str(dat)

# Separate groups
ASD <- subset(dat, KBeein == 1)
TD <- subset(dat, KBeein == 0)

library(psych)
#########################
### Sample description ###
#########################
library(psych)
describeBy(dat$KAlter_Gesamtmonate, dat$KBeein)
xtabs(~dat$KBeein+dat$Kgesch_code)
xtabs(~dat$K_Beein_alle+dat$KBeein)
xtabs(~dat$EBezug+dat$KBeein)
describeBy(dat$EAlter, dat$KBeein)
xtabs(~dat$EBeein+dat$KBeein)
xtabs(~dat$`EBeein[other]`+dat$KBeein)
xtabs(~dat$`EBildu[SQ001]`+dat$KBeein)
xtabs(~dat$`EBildu[SQ002]`+dat$KBeein)
xtabs(~dat$KSprech+dat$KBeein)
xtabs(~dat$KGeschw+dat$KBeein)
xtabs(~dat$`KGeschw[other]`+dat$KBeein)

#############################
### Descriptiv Supplement ###
#############################

### Digital Devices ###
# PC/Laptop
xtabs(~dat$`HaNu[Geraet1][1]`+ dat$KBeein)
xtabs(~ASD$`HaNu[Geraet1][2]`+ ASD$`HaNu[Geraet1][1]`)
xtabs(~TD$`HaNu[Geraet1][2]`+ TD$`HaNu[Geraet1][1]`)
# Tablet
xtabs(~dat$`HaNu[Geraet2][1]`+ dat$KBeein)
xtabs(~ASD$`HaNu[Geraet2][2]`+ ASD$`HaNu[Geraet2][1]`)
xtabs(~TD$`HaNu[Geraet2][2]`+ TD$`HaNu[Geraet2][1]`)
# Smartphone
xtabs(~dat$`HaNu[Geraet3][1]`+ dat$KBeein)
xtabs(~ASD$`HaNu[Geraet3][2]`+ ASD$`HaNu[Geraet3][1]`)
xtabs(~TD$`HaNu[Geraet3][2]`+ TD$`HaNu[Geraet3][1]`)
# Gaming console
xtabs(~dat$`HaNu[Geraet4][1]`+ dat$KBeein)
xtabs(~ASD$`HaNu[Geraet4][2]`+ ASD$`HaNu[Geraet4][1]`)
xtabs(~TD$`HaNu[Geraet4][2]`+ TD$`HaNu[Geraet4][1]`)
# Television
xtabs(~dat$`HaNu[Geraet5][1]`+ dat$KBeein)
xtabs(~ASD$`HaNu[Geraet5][2]`+ ASD$`HaNu[Geraet5][1]`)
xtabs(~TD$`HaNu[Geraet5][2]`+ TD$`HaNu[Geraet5][1]`)
# Radio/Music recorder (Tonieboxen)
xtabs(~dat$`HaNu[Geraet6][1]`+ dat$KBeein)
xtabs(~ASD$`HaNu[Geraet6][2]`+ ASD$`HaNu[Geraet6][1]`)
xtabs(~TD$`HaNu[Geraet6][2]`+ TD$`HaNu[Geraet6][1]`)
# Digital assistants (Alexa)
xtabs(~dat$`HaNu[Geraet7][1]`+ dat$KBeein)
xtabs(~ASD$`HaNu[Geraet7][2]`+ ASD$`HaNu[Geraet7][1]`)
xtabs(~TD$`HaNu[Geraet7][2]`+ TD$`HaNu[Geraet7][1]`)
# Smart-Toys
xtabs(~dat$`HaNu[Geraet8][1]`+ dat$KBeein)
xtabs(~ASD$`HaNu[Geraet8][2]`+ ASD$`HaNu[Geraet8][1]`)
xtabs(~TD$`HaNu[Geraet8][2]`+ TD$`HaNu[Geraet8][1]`)

### Own property ###
# Smartphone
xtabs(~dat$`EigGer[SQ001]`+ dat$KBeein)
# Computer/ Laptop
```

```
xtabs(~dat$`EigGer[SQ002]`+ dat$KBeein)
# Tablet / iPad
xtabs(~dat$`EigGer[SQ003]`+ dat$KBeein)
# Television
xtabs(~dat$`EigGer[SQ004]`+ dat$KBeein)
# Gaming concsole
xtabs(~dat$`EigGer[SQ005]`+ dat$KBeein)

### Digital devices available and usable in the children's room ###
xtabs(~dat$KiZim+ dat$KBeein)

### Average daily time per week ###
# PC / Laptop
xtabs(~dat$`Zeit[Geraet1][1]` + dat$KBeein)
# Tablet
xtabs(~dat$`Zeit[Geraet2][1]` + dat$KBeein)
# Smartphone
xtabs(~dat$`Zeit[Geraet3][1]` + dat$KBeein)
# Gaming console
xtabs(~dat$`Zeit[Geraet4][1]` + dat$KBeein)
# Music recorder (including Tonieboxes, audio plays)
xtabs(~dat$`Zeit[Geraet5][1]` + dat$KBeein)
# Television (including streaming services such as Netflix, Disney+)
xtabs(~dat$`Zeit[Geraet6][1]` + dat$KBeein)

### Average daily time per weekends/holidays ###
# PC / Laptop
xtabs(~dat$`Zeit[Geraet1][2]` + dat$KBeein)
# Tablet
xtabs(~dat$`Zeit[Geraet2][2]` + dat$KBeein)
# Smartphone
xtabs(~dat$`Zeit[Geraet3][2]` + dat$KBeein)
# Gaming console
xtabs(~dat$`Zeit[Geraet4][2]` + dat$KBeein)
# Music recorder (including Tonieboxes, audio plays)
xtabs(~dat$`Zeit[Geraet5][2]` + dat$KBeein)
# Television (including streaming services such as Netflix, Disney+)
xtabs(~dat$`Zeit[Geraet6][2]` + dat$KBeein)

### Age of daily media use ###
xtabs(~dat$Altreg_code + dat$KBeein)
xtabs(~ASD$Altreg_Zahl + ASD$Altreg_code)
describeBy(ASD$Altreg_Zahl, ASD$Altreg_code)
xtabs(~TD$Altreg_Zahl + TD$Altreg_code)
describeBy(TD$Altreg_Zahl, TD$Altreg_code)

### Maximum media time per day during the week ###
xtabs(~dat$MeZeitTag_Wo_Minuten + dat$KBeein)
describeBy(dat$MeZeitTag_Wo_Minuten, dat$KBeein)
### Maximum media time per day during the weekend ###
xtabs(~dat$MeZeitTag_WoEnd_Minuten + dat$KBeein)
describeBy(dat$MeZeitTag_WoEnd_Minuten, dat$KBeein)

### What children do when they use media
# Entertainment games
xtabs(~dat$`GruNut[Grund1]` + dat$KBeein)
# Educational games
xtabs(~dat$`GruNut[Grund2]` + dat$KBeein)
# View photos
xtabs(~dat$`GruNut[Grund3]` + dat$KBeein)
# Take photos
xtabs(~dat$`GruNut[Grund4]` + dat$KBeein)
# Listen to music/audio plays (including Tonieboxes)
xtabs(~dat$`GruNut[Grund5]` + dat$KBeein)
# Watch movies/videos
xtabs(~dat$`GruNut[Grund6]` + dat$KBeein)
# Programming
xtabs(~dat$`GruNut[Grund7]` + dat$KBeein)
# Chat/talk with others
```

```
xtabs(~dat$`GruNut[Grund8]` + dat$KBeein)

### To what extent parents are aware of their child's media use ###
xtabs(~dat$MitbekNu + dat$KBeein)

### Situations in which media are provided ###
# To bridge waiting times
xtabs(~dat$`SituaGer[SQ001]` + dat$KBeein)
# When my child is bored
xtabs(~dat$`SituaGer[SQ002]` + dat$KBeein)
# To support learning
xtabs(~dat$`SituaGer[SQ003]` + dat$KBeein)
# To have time for other things
xtabs(~dat$`SituaGer[SQ004]` + dat$KBeein)
# As family time together
xtabs(~dat$`SituaGer[SQ005]` + dat$KBeein)
# If my child is not feeling well
xtabs(~dat$`SituaGer[SQ006]` + dat$KBeein)
# When you have reached your limits
xtabs(~dat$`SituaGer[SQ007]` + dat$KBeein)
# To carry out household chores undisturbed
xtabs(~dat$`SituaGer[SQ008]` + dat$KBeein)
# As a reward
xtabs(~dat$`SituaGer[SQ009]` + dat$KBeein)
# Other
xtabs(~dat$SituaGer_Code + dat$KBeein) # Mostly 3 indicated # Regulation applies if my
child is not well

### Rules for media use ###
xtabs(~dat$Regeln + dat$KBeein)


### How many hours can they cope without media ###
xtabs(~dat$Aushalohn + dat$KBeein)

### Are media dispensable? ###
xtabs(~dat$NotweNu + dat$KBeein)

##################
### Subscales ####
# The child's use of digital media in everyday life
dat$U_Mediensucht <- rowSums(dat[, c("UmgangM5", "UmgangI2", "UmgangM3", "UmgangV3",
"UmgangM4",
                                     "UmgangI1", "UmgangM6", "UmgangF3", "UmgangM7",
"UmgangM1",
                                     "UmgangF4", "UmgangM2", "UmgangM8", "UmgangV1",
"UmgangF2")])
dat$U_Mediensucht_m <- rowMeans(dat[, c("UmgangM5", "UmgangI2", "UmgangM3", "UmgangV3",
"UmgangM4",
                                        "UmgangI1", "UmgangM6", "UmgangF3", "UmgangM7",
"UmgangM1",
                                        "UmgangF4", "UmgangM2", "UmgangM8", "UmgangV1",
"UmgangF2")])
alpha(dat[, c("UmgangM5", "UmgangI2", "UmgangM3", "UmgangV3", "UmgangM4", "UmgangI1",
"UmgangM6", "UmgangF3",
              "UmgangM7", "UmgangM1", "UmgangF4", "UmgangM2", "UmgangM8", "UmgangV1",
"UmgangF2")])
dat$U_Medienkompetenz <- rowSums(dat[, c("UmgangS2", "UmgangS4", "UmgangS3",
"UmgangS1")])
dat$U_Medienkompetenz_m <- rowMeans(dat[, c("UmgangS2", "UmgangS4", "UmgangS3",
"UmgangS1")])
alpha(dat[, c("UmgangS2", "UmgangS4", "UmgangS3", "UmgangS1")])
dat$U_EinschraenkungRegulierung <- rowSums(dat[, c("UmgangE2R", "UmgangE1","UmgangE3R",
"UmgangV2","UmgangF1")])
dat$U_EinschraenkungRegulierung_m <- rowMeans(dat[, c("UmgangE2R",
"UmgangE1","UmgangE3R", "UmgangV2","UmgangF1")])
alpha(dat[, c("UmgangE2R", "UmgangE1","UmgangE3R", "UmgangV2","UmgangF1")])

# Parents' concerns about their children's media use
```

```
dat$S_Mediensucht <- rowSums(dat[, c("SorgenS1", "SorgenS2", "SorgenS3")])
dat$S_Mediensucht_m <- rowMeans(dat[, c("SorgenS1", "SorgenS2", "SorgenS3")])
alpha(dat[, c("SorgenS1", "SorgenS2", "SorgenS3")])
dat$S_RealeWeltVerlust<- rowSums(dat[, c("SorgenRW2", "SorgenRW4", "SorgenRW3",
"SorgenRW1")])
dat$S_RealeWeltVerlust_m <- rowMeans(dat[, c("SorgenRW2", "SorgenRW4", "SorgenRW3",
"SorgenRW1")])
alpha(dat[, c("SorgenRW2", "SorgenRW4", "SorgenRW3", "SorgenRW1")])
dat$S_NegAuswirkungGuV <- rowSums(dat[, c("SorgenK1", "SorgenK3","SorgenK2",
"SorgenK4","SorgenRWL5",
                                          "SorgenK5", "SorgenK6")])
dat$S_NegAuswirkungGuV_m <- rowMeans(dat[, c("SorgenK1", "SorgenK3","SorgenK2",
"SorgenK4","SorgenRWL5",
                                             "SorgenK5", "SorgenK6")])
alpha(dat[, c("SorgenK1", "SorgenK3","SorgenK2", "SorgenK4","SorgenRWL5", "SorgenK5",
"SorgenK6")])
dat$S_KontrollverlustKompetenz <- rowSums(dat[, c("SorgenI3", "SorgenI1","SorgenI4",
"SorgenI2","SorgenI5",
                                                  "SorgenI6", "SorgenO1", "SorgenO2")])
dat$S_KontrollverlustKompetenz_m <- rowMeans(dat[, c("SorgenI3", "SorgenI1","SorgenI4",
"SorgenI2","SorgenI5",
                                                     "SorgenI6", "SorgenO1",
"SorgenO2")])
alpha(dat[, c("SorgenI3", "SorgenI1","SorgenI4", "SorgenI2","SorgenI5", "SorgenI6",
"SorgenO1", "SorgenO2")])
dat$S_Sorgengrund <- rowSums(dat[, c("SorgenU1", "SorgenU2")])
dat$S_Sorgengrund_m <- rowMeans(dat[, c("SorgenU1", "SorgenU2")])
alpha(dat[, c("SorgenU1", "SorgenU2")])
dat$Sorgen <- rowSums(dat[, c("SorgenS1", "SorgenS2", "SorgenS3", "SorgenRW2",
"SorgenRW4", "SorgenRW3", "SorgenRW1",
                              "SorgenK1", "SorgenK3","SorgenK2",
"SorgenK4","SorgenRWL5", "SorgenK5", "SorgenK6",
                              "SorgenI3", "SorgenI1","SorgenI4", "SorgenI2","SorgenI5",
"SorgenI6", "SorgenO1", "SorgenO2")])
dat$Sorgen_m <- rowMeans(dat[, c("SorgenS1", "SorgenS2", "SorgenS3", "SorgenRW2",
"SorgenRW4", "SorgenRW3", "SorgenRW1",
                                 "SorgenK1", "SorgenK3","SorgenK2",
"SorgenK4","SorgenRWL5", "SorgenK5", "SorgenK6",
                                 "SorgenI3", "SorgenI1","SorgenI4",
"SorgenI2","SorgenI5", "SorgenI6", "SorgenO1", "SorgenO2")])
alpha(dat[, c("SorgenS1", "SorgenS2", "SorgenS3", "SorgenRW2", "SorgenRW4",
"SorgenRW3", "SorgenRW1",
              "SorgenK1", "SorgenK3","SorgenK2", "SorgenK4","SorgenRWL5", "SorgenK5",
"SorgenK6",
              "SorgenI3", "SorgenI1","SorgenI4", "SorgenI2","SorgenI5", "SorgenI6",
"SorgenO1", "SorgenO2")])

###########################
### Analysis Supplement ###
###########################
### PC/Laptop
shapiro.test(dat$KBeein)
shapiro.test(dat$`HaNu[Geraet1][2]`)
hist(dat$`HaNu[Geraet1][2]`)
library(car)
leveneTest(`HaNu[Geraet1][2]` ~ Group, data = dat)
wilcox.test(dat$`HaNu[Geraet1][2]` ~ dat$KBeein, exact=FALSE)
qnorm(0.4838/2)
describeBy(dat$`HaNu[Geraet1][2]`, dat$KBeein)

### Tablet
shapiro.test(dat$KBeein)
shapiro.test(dat$`HaNu[Geraet2][2]`)
hist(dat$`HaNu[Geraet2][2]`)
library(car)
leveneTest(`HaNu[Geraet2][2]` ~ Group, data = dat)
wilcox.test(dat$`HaNu[Geraet2][2]` ~ dat$KBeein, exact=FALSE)
qnorm(0.01625/2)
describeBy(dat$`HaNu[Geraet2][2]`, dat$KBeein)
```

```
### Smartphone
shapiro.test(dat$KBeein)
shapiro.test(dat$`HaNu[Geraet3][2]`)
hist(dat$`HaNu[Geraet3][2]`)
library(car)
leveneTest(`HaNu[Geraet3][2]` ~ Group, data = dat)
wilcox.test(dat$`HaNu[Geraet3][2]` ~ dat$KBeein, exact=FALSE)
qnorm(0.0121/2)
describeBy(dat$`HaNu[Geraet3][2]`, dat$KBeein)

### Gaming console
shapiro.test(dat$KBeein)
shapiro.test(dat$`HaNu[Geraet4][2]`)
hist(dat$`HaNu[Geraet4][2]`)
library(car)
leveneTest(`HaNu[Geraet4][2]` ~ Group, data = dat)
wilcox.test(dat$`HaNu[Geraet4][2]` ~ dat$KBeein, exact=FALSE)
qnorm(0.1763/2)
describeBy(dat$`HaNu[Geraet4][2]`, dat$KBeein)

### Television
shapiro.test(dat$KBeein)
shapiro.test(dat$`HaNu[Geraet5][2]`)
hist(dat$`HaNu[Geraet5][2]`)
library(car)
leveneTest(`HaNu[Geraet5][2]` ~ Group, data = dat)
wilcox.test(dat$`HaNu[Geraet5][2]` ~ dat$KBeein, exact=FALSE)
qnorm(0.9305/2)
describeBy(dat$`HaNu[Geraet5][2]`, dat$KBeein)

### Radio/CD player
shapiro.test(dat$KBeein)
shapiro.test(dat$`HaNu[Geraet6][2]`)
hist(dat$`HaNu[Geraet6][2]`)
library(car)
leveneTest(`HaNu[Geraet6][2]` ~ Group, data = dat)
wilcox.test(dat$`HaNu[Geraet6][2]` ~ dat$KBeein, exact=FALSE)
qnorm(0.136/2)
describeBy(dat$`HaNu[Geraet6][2]`, dat$KBeein)

### digital assistant
shapiro.test(dat$KBeein)
shapiro.test(dat$`HaNu[Geraet7][2]`)
hist(dat$`HaNu[Geraet7][2]`)
library(car)
leveneTest(`HaNu[Geraet7][2]` ~ Group, data = dat)
wilcox.test(dat$`HaNu[Geraet7][2]` ~ dat$KBeein, exact=FALSE)
qnorm(0.7954/2)
describeBy(dat$`HaNu[Geraet7][2]`, dat$KBeein)

### SMART-Toys
shapiro.test(dat$KBeein)
shapiro.test(dat$`HaNu[Geraet8][2]`)
hist(dat$`HaNu[Geraet8][2]`)
library(car)
leveneTest(`HaNu[Geraet8][2]` ~ Group, data = dat)
wilcox.test(dat$`HaNu[Geraet8][2]` ~ dat$KBeein, exact=FALSE)
qnorm(0.1507/2)
describeBy(dat$`HaNu[Geraet8][2]`, dat$KBeein)

## How many digital media devices are there in the household?
dat$Anzahl_digMed<-rowSums(subset(dat, select= c(`HaNu[Geraet1][1]`, `HaNu[Geraet2]
[1]`, `HaNu[Geraet3][1]`,`HaNu[Geraet4][1]`,
                                                  `HaNu[Geraet5][1]`, `HaNu[Geraet6]
[1]`, `HaNu[Geraet7][1]`,
                                                  `HaNu[Geraet8][1]`)), na.rm=TRUE)
table(dat$Anzahl_digMed)
shapiro.test(dat$Anzahl_digMed)
```

```
wilcox.test(dat$Anzahl_digMed ~ dat$KBeein, exact=FALSE)
qnorm(0.3789/2) # z=-0.8799249
describeBy(dat$Anzahl_digMed, dat$KBeein)
AnzahlMedien <- wilcox.test(dat$Anzahl_digMed ~ dat$KBeein, exact=FALSE)

# Frequency of media use #
dat$Nutzung<- rowMeans(subset(dat,select = c(`HaNu[Geraet1][2]`, `HaNu[Geraet2][2]`,
`HaNu[Geraet3][2]`,`HaNu[Geraet4][2]`,
                                               `HaNu[Geraet5][2]`, `HaNu[Geraet6][2]`,
`HaNu[Geraet7][2]`,
                                               `HaNu[Geraet8][2]`)), na.rm=TRUE)
table(dat$Nutzung)
shapiro.test(dat$Nutzung)
hist(dat$Nutzung)
leveneTest(Nutzung ~ Group, data = dat)
t.test(dat$Nutzung ~ dat$KBeein, exact=FALSE)
wilcox.test(dat$Nutzung ~ dat$KBeein, exact=FALSE)
qnorm(0.06941/2)
describeBy(dat$Nutzung, dat$KBeein)
HaufigkeitMedien <- wilcox.test(dat$Nutzung ~ dat$KBeein, exact=FALSE)

####################################
### Analyses Mann-Whitney U-test ###
####################################
library(psych)
describeBy(dat$`SorgenK5`, dat$KBeein)
describeBy(dat$`SorgenK6`, dat$KBeein)

# Items: Parental concerns media use and ASD
shapiro.test(ASD$`SorgenK5`)
hist(ASD$`SorgenK5`)
shapiro.test(ASD$`SorgenK6`)
hist(ASD$`SorgenK6`)
shapiro.test(TD$`SorgenK5`)
hist(TD$`SorgenK5`)
shapiro.test(TD$`SorgenK6`)
hist(TD$`SorgenK6`)
shapiro.test(dat$`SorgenK5`)
shapiro.test(dat$`SorgenK6`)
library(car)
leveneTest(`SorgenK5` ~ Group, data = dat)
leveneTest(`SorgenK6` ~ Group, data = dat)
wilcox.test(`SorgenK5` ~ Group, data = dat)
wilcox.test(`SorgenK6` ~ Group, data = dat)
qnorm(0.1195/2)
qnorm(0.0745/2)

## Items together: Parental concerns media use and ASD
dat$S_ASS <- rowMeans(dat[, c("SorgenK5", "SorgenK6")])
alpha(dat[, c("SorgenK5", "SorgenK6")])
shapiro.test(dat$S_ASS)
hist(dat$S_ASS)
leveneTest(S_ASS ~ Group, data = dat)
wilcox.test(S_ASS ~ Group, data = dat)
qnorm(0.04099/2)
describeBy(dat$S_ASS, dat$KBeein)
ASS_Symptome <- wilcox.test(S_ASS ~ Group, data = dat)

### Maximum media time ###
shapiro.test(ASD$MeZeitTag_Wo_Minuten)
hist(ASD$MeZeitTag_Wo_Minuten)
shapiro.test(ASD$MeZeitTag_WoEnd_Minuten)
hist(ASD$MeZeitTag_WoEnd_Minuten)
shapiro.test(TD$MeZeitTag_Wo_Minuten)
hist(TD$MeZeitTag_Wo_Minuten)
shapiro.test(TD$MeZeitTag_WoEnd_Minuten)
hist(TD$MeZeitTag_WoEnd_Minuten)
library(car)
leveneTest(MeZeitTag_Wo_Minuten ~ Group, data = dat)
```

```
leveneTest(MeZeitTag_WoEnd_Minuten ~ Group, data = dat)
wilcox.test(MeZeitTag_Wo_Minuten ~ Group, data = dat)
wilcox.test(MeZeitTag_WoEnd_Minuten ~ Group, data = dat)
qnorm(0.0000006181/2)
qnorm(0.00002906/2)
MaxMeZeit_Wo <- wilcox.test(MeZeitTag_Wo_Minuten ~ Group, data = dat)
MaxMeZeit_WoEnd <- wilcox.test(MeZeitTag_WoEnd_Minuten ~ Group, data = dat)
describeBy(dat$MeZeitTag_Wo_Minuten, dat$Group)
4713.5/(115*56)
describeBy(dat$MeZeitTag_WoEnd_Minuten, dat$Group)
4292/(112*55)


# Subset only maximum media time
datMax<-subset(dat, select= c(MeZeitTag_Wo_Minuten, MeZeitTag_WoEnd_Minuten))
library(reshape2)
datMax_long <- melt(data=datMax,
                    measure.vars = c("MeZeitTag_Wo_Minuten",
"MeZeitTag_WoEnd_Minuten"),
                    value.name = "minuten",
                    variable.name = "Zeitpunkt")
wilcox.test(minuten ~ Zeitpunkt, data = datMax_long)
qnorm(0.0000000000001371/2)
MaxZeit_inWovsWoEnd <- wilcox.test(minuten ~ Zeitpunkt, data = datMax_long)
describeBy(datMax_long$minuten, datMax_long$Zeitpunkt)
7705.5/(171*167)


dat$MeZeitTag_mean <- rowMeans(dat[, c("MeZeitTag_Wo_Minuten",
"MeZeitTag_WoEnd_Minuten")])
str(dat$MeZeitTag_mean)
wilcox.test(MeZeitTag_mean ~ Group, data = dat)
qnorm(0.000001903/2)
describeBy(dat$MeZeitTag_mean , dat$KBeein)
MaxMeZeit_m <- wilcox.test(MeZeitTag_mean ~ Group, data = dat)
describeBy(dat$MeZeitTag_mean, dat$Group)
4475/(112*55)



### How many hours can the child cope without media ###
shapiro.test(ASD$Aushalohn)
hist(ASD$Aushalohn)
shapiro.test(TD$Aushalohn)
hist(TD$Aushalohn)
library(car)
leveneTest(Aushalohn ~ Group, data = dat)
wilcox.test(Aushalohn ~ Group, data = dat)
qnorm(0.0000007173/2)
describeBy(dat$Aushalohn, dat$KBeein)
AushaltohnMed <- wilcox.test(Aushalohn ~ Group, data = dat)
describeBy(dat$Aushalohn, dat$Group)
1884/(117*58)



### Group comparison ###
## The child's use of digital media in everyday life  ##
shapiro.test(dat$KBeein)
hist(dat$KBeein)
# Preference for digital media and media addiction #
shapiro.test(dat$U_Mediensucht_m)
hist(dat$U_Mediensucht_m)
leveneTest(U_Mediensucht_m ~ Group, data = dat)
wilcox.test(U_Mediensucht_m ~ Group, data = dat)
qnorm(0.0000005427/2)
describeBy(dat$U_Mediensucht_m, dat$KBeein)
U_mediaaddition<- wilcox.test(U_Mediensucht_m ~ Group, data = dat)
describeBy(dat$U_Mediensucht_m, dat$Group)
```

```
4893.5/(115*58)
# Media skills  #
shapiro.test(dat$U_Medienkompetenz_m)
hist(dat$U_Medienkompetenz_m)
leveneTest(U_Medienkompetenz_m ~ Group, data = dat)
wilcox.test(U_Medienkompetenz_m ~ Group, data = dat)
qnorm(0.000758/2)
describeBy(dat$U_Medienkompetenz_m, dat$KBeein)
U_mediakompetenz <- wilcox.test(U_Medienkompetenz_m ~ Group, data = dat)
describeBy(dat$U_Mediensucht_m, dat$Group)
4348/(115*58)
# Restrictions and challenges in regulating media use
shapiro.test(dat$U_EinschraenkungRegulierung_m)
hist(dat$U_EinschraenkungRegulierung_m)
leveneTest(U_EinschraenkungRegulierung_m ~ Group, data = dat)
wilcox.test(U_EinschraenkungRegulierung_m ~ Group, data = dat)
qnorm(0.0000000001376/2)
describeBy(dat$U_EinschraenkungRegulierung_m, dat$KBeein)
EinschreankungRegu <- wilcox.test(U_EinschraenkungRegulierung_m ~ Group, data = dat)
describeBy(dat$U_EinschraenkungRegulierung_m, dat$Group)
5248.5/(115*57)


## Parents' concerns about their children's media use ##
# Media addiction #
shapiro.test(dat$S_Mediensucht_m)
hist(dat$S_Mediensucht_m)
leveneTest(S_Mediensucht_m ~ Group, data = dat)
wilcox.test(S_Mediensucht_m ~ Group, data = dat)
qnorm(0.00001812/2)
describeBy(dat$S_Mediensucht_m, dat$KBeein)
SorgenMediensucht <- wilcox.test(S_Mediensucht_m ~ Group, data = dat)
describeBy(dat$S_Mediensucht_m, dat$Group)
4743.5/(117*58)
# Loss of connection to the real world #
shapiro.test(dat$S_RealeWeltVerlust_m)
hist(dat$S_RealeWeltVerlust_m)
leveneTest(S_RealeWeltVerlust_m ~ Group, data = dat)
wilcox.test(S_RealeWeltVerlust_m ~ Group, data = dat)
qnorm(0.00507/2)
describeBy(dat$S_RealeWeltVerlust_m, dat$KBeein)
VerlustrealWelt <- wilcox.test(S_RealeWeltVerlust_m ~ Group, data = dat)
describeBy(dat$S_RealeWeltVerlust_m, dat$Group)
4203/(115*58)
# Negative effects on health and behavior  #
shapiro.test(dat$S_NegAuswirkungGuV_m)
hist(dat$S_NegAuswirkungGuV_m)
leveneTest(S_NegAuswirkungGuV_m ~ Group, data = dat)
wilcox.test(S_NegAuswirkungGuV_m ~ Group, data = dat)
qnorm(0.001447/2)
describeBy(dat$S_NegAuswirkungGuV_m, dat$KBeein)
SorgenGesundheit <- wilcox.test(S_NegAuswirkungGuV_m ~ Group, data = dat)
describeBy(dat$S_NegAuswirkungGuV_m, dat$Group)
3683.5/(99*57)
# Worries about online dangers, loss of control, and parental media skills #
shapiro.test(dat$S_KontrollverlustKompetenz_m)
hist(dat$S_KontrollverlustKompetenz_m)
leveneTest(S_KontrollverlustKompetenz_m ~ Group, data = dat)
wilcox.test(S_KontrollverlustKompetenz_m ~ Group, data = dat)
qnorm(0.1414/2)
describeBy(dat$S_KontrollverlustKompetenz_m, dat$KBeein)
# No support as a reason for worries
describeBy(dat$S_Sorgengrund_m, dat$KBeein)
#  Overall: concerns #
shapiro.test(dat$Sorgen_m)
hist(dat$Sorgen_m)
leveneTest(Sorgen_m ~ Group, data = dat)
wilcox.test(Sorgen_m ~ Group, data = dat)
qnorm(0.0009639/2)
```

```
describeBy(dat$Sorgen_m, dat$KBeein)
Sorgen <- wilcox.test(Sorgen_m ~ Group, data = dat)
describeBy(dat$Sorgen_m, dat$Group)
3647/(97*57)

## Concerns and sex #
shapiro.test(dat$Kgesch_code)
hist(dat$Kgesch_code)
shapiro.test(dat$Sorgen_m)
hist(dat$Sorgen_m)
library(car)
leveneTest(Sorgen_m ~ KGesch, data = dat)
wilcox.test(Sorgen_m ~ KGesch, data = dat)
qnorm(0.2136/2)
describeBy(dat$Sorgen_m, dat$KGesch)
SorgenGeschlecht <- wilcox.test(Sorgen_m ~ KGesch, data = dat)

## If my child is not doing well, they get media: groups ##
shapiro.test(dat$`SituaGer[SQ006]`)
hist(dat$`SituaGer[SQ006]`)
shapiro.test(dat$KBeein)
hist(dat$KBeein)
library(car)
leveneTest(`SituaGer[SQ006]` ~ Group, data = dat)
wilcox.test(`SituaGer[SQ006]` ~ Group, data = dat)
qnorm(0.00002217/2)
describeBy(dat$`SituaGer[SQ006]`, dat$KBeein)
schwierigesTemperament <- wilcox.test(`SituaGer[SQ006]` ~ Group, data = dat)
describeBy(dat$`SituaGer[SQ006]`, dat$Group)
4571/(116*57)

### Final Bonferroni correction for group comparisons ####
# Mann-Whitney U-test
p_values <- c(AnzahlMedien$p.value,
              HaufigkeitMedien$p.value,
              AushaltohnMed$p.value,
              MaxMeZeit_Wo$p.value,
              MaxMeZeit_WoEnd$p.value,
              MaxZeit_inWovsWoEnd$p.value,
              MaxMeZeit_m$p.value,
              EinschreankungRegu$p.value,
              schwierigesTemperament$p.value,
              U_mediakompetenz$p.value,
              U_mediaaddition$p.value,
              ASS_Symptome$p.value,
              SorgenGesundheit$p.value,
              VerlustrealWelt$p.value,
              Sorgen$p.value,
              SorgenMediensucht$p.value,
              SorgenGeschlecht$p.value
)
# Bonferroni-correction
p.adjust(p_values, method = "bonferroni")
adjusted_p_values <- p.adjust(p_values, method = "bonferroni")

# Display results
data.frame(Test = c("Anzahl Medien", "Haufigkeit Medien", "Aushalten ohne Medien", "Max medienzeit inWo", "Max Medienzeit WoEnd",
                    "MaximaleMedienzeit InWovsWoEnd", "Max Medienzeit mean", "EinschraenkungRegulierung", "schwieriges Temperament",
                    "U_Medienkompetenz", "Medien addiction", "ASS Symptome", "Sorgen Gesundheit", "Verlust reale Welt",
                    "Sorgen", "Sorgen Mediensucht", "Sorgen vs. Geschlecht"),
           P_Wert = p_values,
           Angepasster_P_Wert = adjusted_p_values)

##############################
### Analyses Correlations ###
##############################
```

```
# Requirements for normal distribution
shapiro.test(dat$U_Mediensucht_m)
shapiro.test(dat$S_Mediensucht_m)
shapiro.test(dat$U_EinschraenkungRegulierung_m)
shapiro.test(dat$U_Medienkompetenz_m)
shapiro.test(dat$S_KontrollverlustKompetenz_m)
shapiro.test(dat$MeZeitTag_mean)
shapiro.test(dat$Sorgen_m)
shapiro.test(dat$KAlter_Gesamtmonate)
shapiro.test(dat$Aushalohn)
shapiro.test(dat$S_NegAuswirkungGuV_m)

## Preference for digital media and media addiction + Media addiction
cor.test(dat$U_Mediensucht_m, dat$S_Mediensucht_m, method = "spearman", use =
"complete.obs")
Medienaddiction_Mediensucht <- cor.test(dat$S_Mediensucht_m, dat$U_Mediensucht_m,
method = "spearman", use = "complete.obs")
## Preference for digital media and media addiction + Negative effects on health and
behavior
cor.test(dat$U_Mediensucht_m, dat$S_NegAuswirkungGuV_m, method = "spearman", use =
"complete.obs")
Mediaaddiction_SorgenGesundheit <- cor.test(dat$U_Mediensucht_m,
dat$S_NegAuswirkungGuV_m, method = "spearman", use = "complete.obs")
## Restrictions and challenges in regulating media use + Media addiction
cor.test(dat$U_EinschraenkungRegulierung_m, dat$S_Mediensucht_m, method = "spearman",
use = "complete.obs")
SorgenMediensucht_ProbEinschraen <- cor.test(dat$S_Mediensucht_m,
dat$U_EinschraenkungRegulierung_m, method = "spearman", use = "complete.obs")
## Media skills + Worries about online dangers, loss of control, and parental media
skills
cor.test(dat$U_Medienkompetenz_m, dat$S_KontrollverlustKompetenz_m, method =
"spearman", use = "complete.obs")
## Maximum media time + Concerns
cor.test(dat$MeZeitTag_mean, dat$Sorgen_m, method = "spearman", use = "complete.obs")
Sorgen_maxMedienzeit <- cor.test(dat$Sorgen_m, dat$MeZeitTag_mean, method = "spearman",
use = "complete.obs")
## Maximum media time + Age
cor.test(dat$MeZeitTag_mean, dat$KAlter_Gesamtmonate, method = "spearman", use =
"complete.obs")
MaxMedienzeit_Alter <- cor.test(dat$MeZeitTag_mean, dat$KAlter_Gesamtmonate, method =
"spearman", use = "complete.obs")
## Maximum media time + Difficulty limiting media use
cor.test(dat$MeZeitTag_mean, dat$U_EinschraenkungRegulierung_m, method = "spearman",
use = "complete.obs")
MaxMedienzeit_ProbEinschraen <- cor.test(dat$MeZeitTag_mean,
dat$U_EinschraenkungRegulierung_m, method = "spearman", use = "complete.obs")
## Maximum media time +  can easily cope without mediae
cor.test(dat$MeZeitTag_mean, dat$Aushalohn, method = "spearman", use = "complete.obs")
MaxMedienzeit_AushaltohnProb <- cor.test(dat$MeZeitTag_mean, dat$Aushalohn, method =
"spearman", use = "complete.obs")
## Concerns + Age
cor.test(dat$Sorgen_m, dat$KAlter_Gesamtmonate, method = "spearman", use =
"complete.obs")
Sorgen_alter <- cor.test(dat$Sorgen_m, dat$KAlter_Gesamtmonate, method = "spearman",
use = "complete.obs")
## Concerns + How many hours can the child cope without media
cor.test(dat$Sorgen_m, dat$Aushalohn, method = "spearman", use = "complete.obs")
plot(dat$Sorgen_m, dat$Aushalohn, xlab = "Sorgen", ylab = "Aushalten")
Sorgen_aushaltohne <- cor.test(dat$Sorgen_m, dat$Aushalohn, method = "spearman", use =
"complete.obs")
## Concerns + Difficulty limiting media use
cor.test(dat$Sorgen_m, dat$U_EinschraenkungRegulierung_m, method = "spearman", use =
"complete.obs")
Sorgen_ProbEinschr <- cor.test(dat$Sorgen_m, dat$U_EinschraenkungRegulierung_m, method
= "spearman", use = "complete.obs")
## Concerns + Media addiction
cor.test(dat$Sorgen_m, dat$U_Mediensucht_m, method = "spearman", use = "complete.obs")
Sorgen_Mediaadditcion <- cor.test(dat$Sorgen_m, dat$U_Mediensucht_m, method =
"spearman", use = "complete.obs")
```

```
## Media addiction + How many hours can the child cope without media
cor.test(dat$S_Mediensucht_m, dat$Aushalohn, method = "spearman", use = "complete.obs")
SorgenMediensucht_Aushaltohn <- cor.test(dat$S_Mediensucht_m, dat$Aushalohn, method =
"spearman", use = "complete.obs")

# Separated by group: Concerns + How many hours can the child cope without media
cor.test(ASD$Sorgen_m, ASD$Aushalohn, method = "spearman", use = "complete.obs")
plot(ASD$Sorgen_m, ASD$Aushalohn, xlab = "Sorgen", ylab = "Aushalten")
cor.test(TD$Sorgen_m, TD$Aushalohn, method = "spearman", use = "complete.obs")
plot(TD$Sorgen_m, TD$Aushalohn, xlab = "Sorgen", ylab = "Aushalten")

### Final correlation Bonferroni correction ####
p_values_cor <- c(Sorgen_ProbEinschr$p.value,
              Sorgen_Mediaadditcion$p.value,
              Sorgen_aushaltohne$p.value,
              Sorgen_maxMedienzeit$p.value,
              Sorgen_alter$p.value,
              MaxMedienzeit_AushaltohnProb$p.value,
              MaxMedienzeit_ProbEinschraen$p.value,
              MaxMedienzeit_Alter$p.value,
              SorgenMediensucht_Aushaltohn$p.value,
              SorgenMediensucht_ProbEinschraen$p.value,
              Mediaaddiction_SorgenGesundheit$p.value,
              Medienaddiction_Mediensucht$p.value)

# Bonferroni correction
adjusted_p_values_cor <- p.adjust(p_values_cor, method = "bonferroni")

# Displays results
data.frame(Korrelation = c("Sorgen_ProbEinschr", "Sorgen_Mediaadditcion",
"Sorgen_aushaltohne", "Sorgen_maxMedienzeit", "Sorgen_alter",
                           "MaxMedienzeit_AushaltohnProb",
"MaxMedienzeit_ProbEinschraen", "MaxMedienzeit_Alter",
                           "SorgenMediensucht_Aushaltohn",
"SorgenMediensucht_ProbEinschraen", "Mediaaddiction_SorgenGesundheit",
                           "Medienaddiction_Mediensucht"),
           P_Wert = p_values_cor,
           Angepasster_P_Wert = adjusted_p_values_cor)

########## Hierarchical regression ####
# Manual filtering for complete cases for specific variables
dat_complete <- dat[complete.cases(dat[c("Sorgen_m", "KAlter_Gesamtmonate",
"MeZeitTag_mean", "Aushalohn",
                                         "UmgangE1", "U_Mediensucht_m", "KBeein",
"KGesch")]), ]
# Basic model with control variables child impairment, age, and sex
modell1 <- lm(Sorgen_m ~ KBeein + KAlter_Gesamtmonate + KGesch, data = dat_complete,
na.action = na.exclude)
summary(modell1)
# Expansion model with difficulties in restricting media consumption
modell2 <- lm(Sorgen_m ~ KBeein + KAlter_Gesamtmonate + KGesch + UmgangE1, data =
dat_complete, na.action = na.exclude)
summary(modell2)
# Expansion model with difficulties in restricting media consumption + Preference for
digital media and media addiction
modell3 <- lm(Sorgen_m ~ KBeein + KAlter_Gesamtmonate + KGesch + UmgangE1 +
U_Mediensucht_m, data = dat_complete, na.action = na.exclude)
summary(modell3)
# Expansion model with difficulties in restricting media consumption + Preference for
digital media and media addiction + How many hours can the child cope without media
modell4 <- lm(Sorgen_m ~ KBeein + KAlter_Gesamtmonate + KGesch + UmgangE1 +
U_Mediensucht_m + Aushalohn, data = dat_complete,
              na.action = na.exclude)
summary(modell4)
# Final model with difficulties in restricting media consumption + Preference for
digital media and media addiction + How many hours can the child cope without media +
Maximum media time
modell5 <- lm(Sorgen_m ~ KBeein + KAlter_Gesamtmonate + KGesch + UmgangE1 +
U_Mediensucht_m + Aushalohn + MeZeitTag_mean, data = dat_complete,
```

```
              na.action = na.exclude)
summary(modell5)
# Display R-squared for each model
r_squared_modell1 <- summary(modell1)$r.squared
r_squared_modell2 <- summary(modell2)$r.squared
r_squared_modell3 <- summary(modell3)$r.squared
r_squared_modell4 <- summary(modell4)$r.squared
r_squared_modell5 <- summary(modell5)$r.squared

cat("R² für Modell 1:", r_squared_modell1, "\n")
cat("R² für Modell 2:", r_squared_modell2, "\n")
cat("R² für Modell 3:", r_squared_modell3, "\n")
cat("R² für Modell 4:", r_squared_modell4, "\n")
cat("R² für Modell 5:", r_squared_modell5, "\n")

# F-test for model comparison
anova(modell1, modell2, modell3, modell4, modell5)


### automatically select significant variables and remove insignificant variables ###
# Complete model
vollmodell <- lm(Sorgen_m ~ KBeein + KAlter_Gesamtmonate + MeZeitTag_mean + Aushalohn +
UmgangE1 + U_Mediensucht_m,
                 data = dat_complete, na.action = na.exclude)

# Step-by-step selection
schritt_modell <- step(vollmodell)
```
