# Supplementary material for: Media use among children with ASD: Perspectives and concerns of parents
Source: PLoS One. 2025 Oct 13;20(10):e0332504. doi: 10.1371/journal.pone.0332504 (PMC12517494; doi:10.1371/journal.pone.0332504)
Supplement: S5 Appendix — (PDF) [file pone.0332504.s005.pdf]

S5 Appendix. Output hierarchical regression analysis

Basic model with control variables child impairment, age, and sex

```
Call:
lm(formula = Sorgen_m ~ KBeein + KAlter_Gesamtmonate + KGesch,
    data = dat_complete, na.action = na.exclude)

Residuals:
    Min      1Q  Median      3Q     Max
-2.4813 -1.4088 -0.4914  1.0368  6.3699

Coefficients:
                     Estimate Std. Error t value Pr(>|t|)
(Intercept)          2.362946   0.865114   2.731   0.0071 **
KBeein               0.668205   0.315566   2.117   0.0359 *
KAlter_Gesamtmonate  0.004370   0.007947   0.550   0.5832
KGeschWeiblich      -0.184778   0.346040  -0.534   0.5942
---
Signif. codes:  0 ‘***’ 0.001 ‘**’ 0.01 ‘*’ 0.05 ‘.’ 0.1 ‘ ’ 1

Residual standard error: 1.807 on 143 degrees of freedom
Multiple R-squared:  0.03897,   Adjusted R-squared:  0.0188
F-statistic: 1.933 on 3 and 143 DF,  p-value: 0.127
```

Expansion model with difficulties in restricting media consumption

```
Call:
lm(formula = Sorgen_m ~ KBeein + KAlter_Gesamtmonate + KGesch +
    UmgangE1, data = dat_complete, na.action = na.exclude)

Residuals:
    Min      1Q  Median      3Q     Max
-2.8259 -1.2089 -0.3385  0.9064  6.6873

Coefficients:
                     Estimate Std. Error t value Pr(>|t|)
(Intercept)          2.750626   0.809524   3.398 0.000882 ***
KBeein               0.347650   0.301330   1.154 0.250553
KAlter_Gesamtmonate -0.005419   0.007676  -0.706 0.481389
KGeschWeiblich      -0.227855   0.322308  -0.707 0.480757
UmgangE1             0.248446   0.051848   4.792 4.12e-06 ***
---
Signif. codes:  0 ‘***’ 0.001 ‘**’ 0.01 ‘*’ 0.05 ‘.’ 0.1 ‘ ’ 1

Residual standard error: 1.682 on 142 degrees of freedom
Multiple R-squared:  0.1727,    Adjusted R-squared:  0.1494
F-statistic: 7.412 on 4 and 142 DF,  p-value: 1.886e-05
```

Expansion model with difficulties in restricting media consumption + Preference for digital media and media addiction

```
Call:
lm(formula = Sorgen_m ~ KBeein + KAlter_Gesamtmonate + KGesch +
    UmgangE1 + U_Mediensucht_m, data = dat_complete, na.action = na.exclude)

Residuals:
    Min      1Q  Median      3Q     Max
-2.1232 -0.8658 -0.1782  0.5098  7.6348

Coefficients:
                     Estimate Std. Error t value Pr(>|t|)
(Intercept)          1.611831   0.707988   2.277   0.0243 *
KBeein              -0.183699   0.267117  -0.688   0.4928
KAlter_Gesamtmonate -0.012112   0.006613  -1.832   0.0691 .
KGeschWeiblich      -0.084908   0.275733  -0.308   0.7586
UmgangE1             0.015545   0.054427   0.286   0.7756
U_Mediensucht_m      0.677830   0.092246   7.348 1.48e-11 ***
---
Signif. codes:  0 '***' 0.001 '**' 0.01 '*' 0.05 '.' 0.1 ' ' 1

Residual standard error: 1.436 on 141 degrees of freedom
Multiple R-squared:  0.4018,    Adjusted R-squared:  0.3806
F-statistic: 18.94 on 5 and 141 DF,  p-value: 2.223e-14
```

Expansion model with difficulties in restricting media consumption + Preference for digital media and media addiction + How many hours can the child cope without media

```
Call:
lm(formula = Sorgen_m ~ KBeein + KAlter_Gesamtmonate + KGesch +
    UmgangE1 + U_Mediensucht_m + Aushalohn, data = dat_complete,
    na.action = na.exclude)

Residuals:
    Min      1Q  Median      3Q     Max
-2.3122 -0.8401 -0.2084  0.4901  7.5143

Coefficients:
                     Estimate Std. Error t value Pr(>|t|)
(Intercept)          3.105969   0.994715   3.122  0.00218 **
KBeein              -0.280698   0.267861  -1.048  0.29648
KAlter_Gesamtmonate -0.013900   0.006588  -2.110  0.03665 *
KGeschWeiblich      -0.090724   0.272422  -0.333  0.73961
UmgangE1            -0.006940   0.054813  -0.127  0.89943
U_Mediensucht_m      0.589317   0.100304   5.875 2.94e-08 ***
Aushalohn           -0.176857   0.083717  -2.113  0.03641 *
---
Signif. codes:  0 '***' 0.001 '**' 0.01 '*' 0.05 '.' 0.1 ' ' 1

Residual standard error: 1.418 on 140 degrees of freedom
Multiple R-squared:  0.4203,    Adjusted R-squared:  0.3954
F-statistic: 16.92 on 6 and 140 DF,  p-value: 1.249e-14
```

Final model with difficulties in restricting media consumption + Preference for digital media and media addiction + How many hours can the child cope without media + Maximum media time

```
Call:
lm(formula = Sorgen_m ~ KBeein + KAlter_Gesamtmonate + KGesch +
    UmgangE1 + U_Mediensucht_m + Aushalohn + MeZeitTag_mean,
    data = dat_complete, na.action = na.exclude)

Residuals:
    Min      1Q  Median      3Q     Max
-2.1187 -0.8087 -0.2433  0.4319  7.4549

Coefficients:
                     Estimate Std. Error t value Pr(>|t|)
(Intercept)          3.173988   0.992400   3.198  0.00171 **
KBeein              -0.166739   0.278894  -0.598  0.55091
KAlter_Gesamtmonate -0.011494   0.006783  -1.694  0.09243 .
KGeschWeiblich      -0.068168   0.271937  -0.251  0.80244
UmgangE1            -0.002929   0.054695  -0.054  0.95737
U_Mediensucht_m      0.585019   0.099998   5.850 3.36e-08 ***
Aushalohn           -0.194010   0.084306  -2.301  0.02286 *
MeZeitTag_mean      -0.002505   0.001777  -1.410  0.16086
---
Signif. codes:  0 ‘***’ 0.001 ‘**’ 0.01 ‘*’ 0.05 ‘.’ 0.1 ‘ ’ 1

Residual standard error: 1.413 on 139 degrees of freedom
Multiple R-squared:  0.4285,    Adjusted R-squared:  0.3997
F-statistic: 14.89 on 7 and 139 DF,  p-value: 2.116e-14
```

Model comparison

```
Analysis of Variance Table

Model 1: Sorgen_m ~ KBeein + KAlter_Gesamtmonate + KGesch
Model 2: Sorgen_m ~ KBeein + KAlter_Gesamtmonate + KGesch + UmgangE1
Model 3: Sorgen_m ~ KBeein + KAlter_Gesamtmonate + KGesch + UmgangE1 +
    U_Mediensucht_m
Model 4: Sorgen_m ~ KBeein + KAlter_Gesamtmonate + KGesch + UmgangE1 +
    U_Mediensucht_m + Aushalohn
Model 5: Sorgen_m ~ KBeein + KAlter_Gesamtmonate + KGesch + UmgangE1 +
    U_Mediensucht_m + Aushalohn + MeZeitTag_mean
  Res.Df    RSS Df Sum of Sq       F    Pr(>F)
1    143 466.87
2    142 401.89  1    64.985 32.5328 6.773e-08 ***
3    141 290.60  1   111.284 55.7105 8.336e-12 ***
4    140 281.63  1     8.978  4.4943   0.03578 *
5    139 277.66  1     3.970  1.9873   0.16086
---
Signif. codes:  0 ‘***’ 0.001 ‘**’ 0.01 ‘*’ 0.05 ‘.’ 0.1 ‘ ’ 1
```
