# Supplementary material for: Media use among children with ASD: Perspectives and concerns of parents
Source: PLoS One. 2025 Oct 13;20(10):e0332504. doi: 10.1371/journal.pone.0332504 (PMC12517494; doi:10.1371/journal.pone.0332504)
Supplement: S1 Table — (PDF) [file pone.0332504.s007.pdf]

S1 Table. Availability of digital media in the home and frequency of use (children with ASD: *n* = 117, TD children: *n* = 58)

Digital media	Group	Available at home	never	a few times a month	several times a week	daily
PC/ Laptop			*n* = 107			
	ASD	91.45% (*n* = 107)	60.75% (*n* = 65)	19.63% (*n* = 21)	7.48% (*n* = 8)	12.15% (*n* = 13)
			n = 58			
	TD	100% (*n* = 58)	65.52% (*n* = 38)	20.69% (*n* = 12)	6.9% (*n* = 4)	6.9% (*n* = 4)
Tablet			*n* = 109			
	ASD	93.16% (*n* = 109)	9.17% (*n* = 10)	15.6% (*n* = 17)	27.52% (*n* = 30)	47.71% (*n* = 52)
			n = 54			
	TD	94.83% (*n* = 55)	11.11% (*n* = 6)	27.78% (*n* = 15)	33.33% (*n* = 18)	27.78% (*n* = 15)
Mobile phone			*n* = 116			
	ASD	99.15% (*n* = 116)	19.83% (*n* = 23)	23.28% (*n* = 27)	18.1% (*n* = 21)	38.79% (*n* = 45)
			n = 58			
	TD	100% (*n* = 58)	32.76% (*n* = 19)	24.14% (*n* = 14)	24.14% (*n* = 14)	19% (*n* = 11)
Game console			*n* = 95			
	ASD	81.2% (*n* = 95)	16.84% (*n* = 16)	36.84% (*n* = 35)	28.42% (*n* = 27)	17.9% (*n* = 17)
			n = 42			
	TD	72.41% (*n* = 42)	16.67% (*n* = 7)	52.38% (*n* = 22)	21.43% (*n* = 9)	9.52% (*n* = 4)
TV			*n* = 108			
	ASD	92.31% (*n* = 108)	9.26% (*n* = 10)	18.52% (*n* = 20)	26.85% (*n* = 29)	45.37% (*n* = 49)
			n = 52			
	TD	89.66% (*n* = 52)	1.92% (*n* = 1)	21.15% (*n* = 11)	38.46% (*n* = 20)	38.46% (*n* = 20)
Radio/ Podcast/ CD player/ Audiobooks			*n* = 101			
	ASD	86.33% (*n* = 101)	19.8% (*n* = 20)	20.79% (*n* = 21)	13.86% (*n* = 14)	45.55% (*n* = 46)
			n = 55			
	TD	94.83% (*n* = 55)	14.55% (*n* = 8)	5.46% (*n* = 3)	27.27% (*n* = 15)	52.73% (*n* = 29)
Digital assistants (e.g., Alexa)			*n* = 63			
	ASD	53.85% (*n* = 63)	26.98% (*n* = 17)	19.05% (*n* = 12)	17.46% (*n* = 11)	36.51% (*n* = 23)
			n = 23			
	TD	41.38% (*n* = 24)	26.09% (*n* = 6)	8.7% (*n* = 2)	30.44% (*n* = 7)	34.78% (*n* = 8)
SMART Toys (web-connected devices)			*n* = 81			
	ASD	69.23% (*n* = 81)	49.38% (*n* = 40)	37.04% (*n* = 30)	12.35% (*n* = 10)	1.24% (*n* = 1)
			n = 38			
	TD	65.52% (*n* = 38)	31.58% (*n* = 12)	55.26% (*n* = 21)	13.16% (*n* = 5)	0% (*n* = 0)
