# Supplementary material for: Media use among children with ASD: Perspectives and concerns of parents
Source: PLoS One. 2025 Oct 13;20(10):e0332504. doi: 10.1371/journal.pone.0332504 (PMC12517494; doi:10.1371/journal.pone.0332504)
Supplement: S2 Table — (PDF) [file pone.0332504.s008.pdf]

S2 Table. Own property of digital media (children with ASD: $n = 117$, TD children: $n = 58$)

Digital media	Group	Own property
PC/ Laptop	ASD	19.66% ($n = 23$)
	TD	8.62% ($n = 5$)
Tablet	ASD	64.1% ($n = 75$)
	TD	37.93% ($n = 22$)
Mobile phone	ASD	34.19% ($n = 40$)
	TD	31.03% ($n = 18$)
Game console	ASD	35.04% ($n = 41$)
	TD	22.41% ($n = 13$)
TV	ASD	18.8% ($n = 22$)
	TD	15.52% ($n = 9$)
