# Supplementary material for: Media use among children with ASD: Perspectives and concerns of parents
Source: PLoS One. 2025 Oct 13;20(10):e0332504. doi: 10.1371/journal.pone.0332504 (PMC12517494; doi:10.1371/journal.pone.0332504)
Supplement: S3 Table — (PDF) [file pone.0332504.s009.pdf]

S3 Table. Devices available and usable in the children's room

**Group**	**Devices available and usable in the children's room**		**We only have one shared living space**
	**Yes**	**No**	
ASD (*n* = 117)	52.14% (*n* = 61)	37.61% (*n* = 44)	10.26% (*n* = 12)
TD (*n* = 58)	48.28% (*n* = 28)	44.83% (*n* = 26)	6.9% (*n* = 4)
