# Supplementary material for: Media use among children with ASD: Perspectives and concerns of parents
Source: PLoS One. 2025 Oct 13;20(10):e0332504. doi: 10.1371/journal.pone.0332504 (PMC12517494; doi:10.1371/journal.pone.0332504)
Supplement: S4 Table — (PDF) [file pone.0332504.s010.pdf]

S4 Table. Average time spent on digital media per day

Digital media	Group	Average time spent on digital media per day …	Never	until up to 30 min	30 min up to 2 hours	2 hours up to 4 hours	4 hours and more
PC/ Laptop	ASD	during the week	63.25% (*n* = 74)	14.53% (*n* = 17)	*n* = 117<br>11.11% (*n* = 13)	7.69% (*n* = 9)	3.42% (*n* = 4)
		on weekends/ holidays	60% (*n* = 69)	13.91% (*n* = 16)	*n* = 115<br>9.57% (*n* = 11)	4.35% (*n* = 5)	12.17% (*n* = 14)
	TD	during the week	74.14% (*n* = 43)	15.52% (*n* = 9)	*n* = 58<br>10.35% (*n* = 6)	0% (*n* = 0)	0% (*n* = 0)
		on weekends/ holidays	67.27% (*n* = 37)	20% (*n* = 11)	*n* = 55<br>9.09% (*n* = 5)	1.82% (*n* = 1)	1.82% (*n* = 1)
Tablet	ASD	during the week	20.51% (*n* = 24)	27.35% (*n* = 32)	*n* = 117<br>29.06% (*n* = 34)	14.53% (*n* = 17)	8.55% (*n* = 10)
		on weekends/ holidays	15.52% (*n* = 18)	16.38% (*n* = 19)	*n* = 116<br>31.04% (*n* = 36)	15.52% (*n* = 18)	21.55% (*n* = 25)
	TD	during the week	31.04% (*n* = 18)	37.93% (*n* = 22)	*n* = 58<br>24.14% (*n* = 14)	6.9% (*n* = 4)	0% (*n* = 0)
		on weekends/ holidays	18.18% (*n* = 10)	34.55% (*n* = 19)	*n* = 55<br>30.91% (*n* = 17)	10.91% (*n* = 6)	5.46% (*n* = 3)
Mobile phone	ASD	during the week	35.9% (*n* = 42)	39.32% (*n* = 46)	*n* = 117<br>12.82% (*n* = 15)	5.13% (*n* = 6)	6.84% (*n* = 8)
		on weekends/ holidays	27.35% (*n* = 32)	33.33% (*n* = 39)	*n* = 117<br>17.95% (*n* = 21)	9.4% (*n* = 11)	11.97% (*n* = 14)
	TD	during the week	43.1% (*n* = 25)	34.48% (*n* = 20)	*n* = 58<br>18.97% (*n* = 11)	3.45% (*n* = 2)	0% (*n* = 0)
		on weekends/ holidays	33.33% (*n* = 18)	33.33% (*n* = 18)	*n* = 54<br>18.52% (*n* = 10)	11.11% (*n* = 6)	3.7% (*n* = 2)
Game console	ASD	during the week	41.88% (*n* = 49)	30.77% (*n* = 36)	*n* = 117<br>23.08% (*n* = 27)	0.86% (*n* = 1)	3.42% (*n* = 4)
		on weekends/ holidays	29.57% (*n* = 34)	24.35% (*n* = 28)	*n* = 115<br>27.83% (*n* = 32)	13.04% (*n* = 15)	5.22% (*n* = 6)
	TD	during the week	63.79% (*n* = 37)	22.41% (*n* = 13)	*n* = 58<br>12.07% (*n* = 7)	1.72% (*n* = 1)	0% (*n* = 0)
		on weekends/ holidays	38.18% (*n* = 21)	18.18% (*n* = 10)	*n* = 55<br>34.55% (*n* = 19)	5.46% (*n* = 3)	3.64% (*n* = 2)

Digital media	Group	Average time spent on digital media per day …	Never	until up to 30 min	30 min up to 2 hours	2 hours up to 4 hours	4 hours and more
TV	ASD				n = 117		
		during the week	25.64% (n = 30)	29.06% (n = 34)	31.62% (n = 37)	9.4% (n = 11)	4.27% (n = 5)
					n = 116		
		on weekends/ holidays	12.93% (n = 15)	13.79% (n = 16)	44.83% (n = 52)	18.97% (n = 22)	9.48% (n = 11)
	TD				n = 56		
		during the week	21.43% (n = 12)	41.07% (n = 23)	33.93% (n = 19)	3.57% (n = 2)	0% (n = 0)
					n = 56		
		on weekends/ holidays	5.36% (n = 3)	14.29% (n = 8)	60.71% (n = 34)	16.07% (n = 9)	3.57% (n = 2)
Music recorder (i.e. Tonie boxes, Audio-books)	ASD				n = 117		
		during the week	29.92% (n = 35)	23.93% (n = 28)	33.33% (n = 39)	8.55% (n = 10)	4.27% (n = 5)
					n = 115		
		on weekends/ holidays	27.83% (n = 32)	23.48% (n = 27)	25.22% (n = 29)	14.78% (n = 17)	8.7% (n = 10)
	TD				n = 58		
		during the week	20.69% (n = 12)	32.76% (n = 19)	41.38% (n = 24)	5.17% (n = 3)	0% (n = 0)
					n = 54		
		on weekends/ holidays	11.11% (n = 6)	24.07% (n = 13)	50% (n = 27)	12.96% (n = 7)	1.85% (n = 1)
