# Supplementary material for: Media use among children with ASD: Perspectives and concerns of parents
Source: PLoS One. 2025 Oct 13;20(10):e0332504. doi: 10.1371/journal.pone.0332504 (PMC12517494; doi:10.1371/journal.pone.0332504)
Supplement: S5 Table — (PDF) [file pone.0332504.s011.pdf]

S5 Table. The maximum amount of media time that parents consider appropriate per day (in min)

Group	maximum amount of media time		
	**during the week**	**on weekends/ holidays**	**averaged over the week and weekend**
ASD	*n* = 115<br>*M* = 120.57 (*SD* = 81.93)<br>Range: 0-420	*n* = 112<br>*M* = 182.54 (*SD* = 92.69)<br>Range: 45-480	*n* = 112<br>*M* = 150.76 (*SD* = 81.23)<br>Range: 30-390
TD	*n* = 56<br>*M* = 63.75 (*SD* = 33.05)<br>Range: 0-120	*n* = 55<br>*M* = 122.27 (*SD* = 58.1)<br>Range: 45-240	N = 55<br>*M* = 93.05 (*SD* = 44)<br>Range: 30-180
