# Supplementary material for: Media use among children with ASD: Perspectives and concerns of parents
Source: PLoS One. 2025 Oct 13;20(10):e0332504. doi: 10.1371/journal.pone.0332504 (PMC12517494; doi:10.1371/journal.pone.0332504)
Supplement: S6 Table — (PDF) [file pone.0332504.s012.pdf]

S6 Table. Age from which the child uses digital media almost daily

	**ASD (*n* = 117)**	**TD (*n* = 58)**
Does not yet use daily	6.84% (*n* = 8)	31.04% (*n* = 18)
- Age at which parents would allow daily media use	- *M* = 10.83 (*SD* = 1.33); Range 10-13; *n* = 6	- *M* = 11.24 (*SD* =1.89); Range 8-14; *n* = 17
Daily use	93.16% (*n* = 109)	68.97% (*n* = 40)
- starting age of daily use	- *M* = 5.38 (*SD* = 2.26); Range 1-12; *n* = 109	- *M* = 5.86 (*SD* = 2); Range 2-10; *n* = 40
