# Supplementary material for: Media use among children with ASD: Perspectives and concerns of parents
Source: PLoS One. 2025 Oct 13;20(10):e0332504. doi: 10.1371/journal.pone.0332504 (PMC12517494; doi:10.1371/journal.pone.0332504)
Supplement: S7 Table — (PDF) [file pone.0332504.s013.pdf]

S7 Table. Reasons for using digital media as named by parents across both groups (children with ASD: *n* = 117, TD children: *n* = 58)

Child uses digital media to…	Group	never	a few times a month	several times a week	daily
play entertain-ment games				*n* = 115	
	ASD	12.17% (*n* = 14)	19.13% (*n* = 22)	32.17% (*n* = 37)	36.52% (*n* = 42)
				n = 56	
	TD	17.86% (*n* = 10)	26.79% (*n* = 15)	28.57% (*n* = 16)	25% (*n* = 14)
play learning games				*n* = 116	
	ASD	13.79% (*n* = 16)	29.31% (*n* = 34)	39.66% (*n* = 46)	17.24% (*n* = 20)
				n = 58	
	TD	6.9% (*n* = 4)	50% (*n* = 29)	36.21% (*n* = 21)	6.9% (*n* = 4)
view photos				*n* = 117	
	ASD	33.33% (*n* = 39)	40.17% (*n* = 47)	20.51% (*n* = 24)	5.98% (*n* = 7)
				n = 57	
	TD	22.81% (*n* = 13)	42.11% (*n* = 24)	28.07% (*n* = 16)	7.02% (*n* = 4)
take photos/ videos				*n* = 117	
	ASD	32.48% (*n* = 38)	45.3% (*n* = 53)	19.66% (*n* = 23)	2.56% (*n* = 3)
				n = 57	
	TD	35.09% (*n* = 20)	38.6% (*n* = 22)	22.81% (*n* = 13)	3.51% (*n* = 2)
listen to music/ podcasts/ audiobooks				*n* = 115	
	ASD	12.17% (*n* = 14)	19.13% (*n* = 22)	15.65% (*n* = 18)	53.04% (*n* = 61)
				n = 58	
	TD	3.45% (*n* = 2)	15.52% (*n* = 9)	25.86% (*n* = 15)	55.17% (*n* = 32)
watch movies/ videos				*n* = 117	
	ASD	4.27% (*n* = 5)	18.8% (*n* = 22)	28.21% (*n* = 33)	48.72% (*n* = 57)
				n = 57	
	TD	10.53% (*n* = 6)	22.81% (*n* = 13)	40.35% (*n* = 23)	26.32% (*n* = 15)
Programming				*n* = 114	
	ASD	76.32% (*n* = 87)	15.79% (*n* = 18)	5.26% (*n* = 6)	2.63% (*n* = 3)
				n = 57	
	TD	94.74% (*n* = 54)	3.51% (*n* = 2)	1.75% (*n* = 1)	0% (*n* = 0)
chat/ interact with others				*n* = 117	
	ASD	66.67% (*n* = 78)	10.26% (*n* = 12)	10.26% (*n* = 12)	12.82% (*n* = 15)
				n = 57	
	TD	61.4% (*n* = 35)	12.28% (*n* = 7)	8.77% (*n* = 5)	17.54% (*n* = 10)
