# Supplementary material for: Media use among children with ASD: Perspectives and concerns of parents
Source: PLoS One. 2025 Oct 13;20(10):e0332504. doi: 10.1371/journal.pone.0332504 (PMC12517494; doi:10.1371/journal.pone.0332504)
Supplement: S8 Table — (PDF) [file pone.0332504.s014.pdf]

S8 Table. Situations in which the child is given a digital device

Situations	Group	never	sometimes	often	regularly
To bridge waiting time	ASD	*n* = 115			
		10.44% (*n* = 12)	48.7% (*n* = 56)	15.65% (*n* = 18)	26.96% (*n* = 31)
	TD	*n* = 56			
		35.71% (*n* = 20)	51.79% (*n* = 29)	12.5% (*n* = 7)	1.79% (*n* = 1)
When my child is bored	ASD	*n* = 116			
		17.24% (*n* = 20)	58.62% (*n* = 68)	11.21% (*n* = 13)	13.79% (*n* = 16)
	TD	*n* = 58			
		43.1% (*n* = 25)	41.38% (*n* = 24)	10.35% (*n* = 6)	1.72% (*n* = 1)
To support learning	ASD	*n* = 117			
		5.98% (*n* = 7)	29.92% (*n* = 35)	34.19% (*n* = 40)	29.92% (*n* = 35)
	TD	*n* = 57			
		10.53% (*n* = 6)	36.84% (*n* = 21)	35.09% (*n* = 20)	17.54% (*n* = 10)
To have time for other things	ASD	*n* = 117			
		15.39% (*n* = 18)	51.28% (*n* = 60)	18.8% (*n* = 22)	14.53% (*n* = 17)
	TD	*n* = 57			
		40.35% (*n* = 23)	49.12% (*n* = 28)	7.02% (*n* = 4)	3.51% (*n* = 2)
As family time together	ASD	*n* = 115			
		30.44% (*n* = 35)	53.04% (*n* = 61)	9.57% (*n* = 11)	8.7% (*n* = 10)
	TD	*n* = 58			
		37.93% (*n* = 22)	48.28% (*n* = 28)	8.62% (*n* = 5)	1.72% (*n* = 1)
When my child is not feeling well	ASD	*n* = 117			
		11.97% (*n* = 14)	29.92% (*n* = 35)	26.5% (*n* = 31)	30.77% (*n* = 36)
	TD	*n* = 57			
		29.83% (*n* = 17)	42.11% (*n* = 24)	21.05% (*n* = 12)	7.02% (*n* = 4)
When your own stress limits have been reached	ASD	*n* = 114			
		11.4% (*n* = 13)	37.72% (*n* = 43)	30.7% (*n* = 35)	22.81% (*n* = 26)
	TD	*n* = 57			
		42.11% (*n* = 24)	40.35% (*n* = 23)	10.53% (*n* = 6)	7.02% (*n* = 4)
to carry out household activities undisturbed	ASD	*n* = 117			
		27.35% (*n* = 32)	39.32% (*n* = 46)	19.66% (*n* = 23)	12.82% (*n* = 15)
	TD	*n* = 57			
		38.6% (*n* = 22)	45.61% (*n* = 26)	10.53% (*n* = 6)	1.75% (*n* = 1)
As a reward	ASD	*n* = 117			
		48.72% (*n* = 57)	39.32% (*n* = 46)	5.98% (*n* = 7)	4.27% (*n* = 5)
	TD	*n* = 57			
		40.35% (*n* = 23)	49.12% (*n* = 28)	10.53% (*n* = 6)	0% (*n* = 0)
