# Supplementary material for: Media use among children with ASD: Perspectives and concerns of parents
Source: PLoS One. 2025 Oct 13;20(10):e0332504. doi: 10.1371/journal.pone.0332504 (PMC12517494; doi:10.1371/journal.pone.0332504)
Supplement: S9 Table — (PDF) [file pone.0332504.s015.pdf]

S9 Table. Parents' awareness of their children's use of digital media

Group	Parents' awareness of their children's use of digital media		
	(almost) not at all	partly	completely
ASD (*n* = 117)	0% (*n* = 0)	12.82% (*n* = 15)	87.18% (*n* = 102)
TD (*n* = 58)	0% (*n* = 0)	18.97% (*n* = 11)	81.04% (*n* = 47)
