# Supplementary material for: Media use among children with ASD: Perspectives and concerns of parents
Source: PLoS One. 2025 Oct 13;20(10):e0332504. doi: 10.1371/journal.pone.0332504 (PMC12517494; doi:10.1371/journal.pone.0332504)
Supplement: S10 Table — (PDF) [file pone.0332504.s016.pdf]

**S10 Table.** Rules for media use

| Group | Existing rules on media use | |
|---|---|---|
| | No | Yes |
| ASD (*n* = 116) | 19.83% (*n* = 23) | 80.17% (*n* = 93) |
| TD (*n* = 57) | 15.53% (*n* = 6) | 89.47% (*n* = 51) |
