# Supplementary material for: Media use among children with ASD: Perspectives and concerns of parents
Source: PLoS One. 2025 Oct 13;20(10):e0332504. doi: 10.1371/journal.pone.0332504 (PMC12517494; doi:10.1371/journal.pone.0332504)
Supplement: S11 Table — (PDF) [file pone.0332504.s017.pdf]

S11 Table. The amount of time that the child can cope without using media in their everyday life

**Time to cope without media**	**ASD (*n* = 117)**	**TD (*n* = 58)**
Not at all	6.84% (*n* = 8)	0% (*n* = 0)
1-2 hours	15.39% (*n* = 18)	6.9% (*n* = 4)
3-4 hours	4.27% (*n* = 5)	0% (*n* = 0)
5-6 hours	9.4% (*n* = 11)	0% (*n* = 0)
7-12 hours	12.82% (*n* = 15)	12.07% (*n* = 7)
A whole day	26.5% (*n* = 31)	18.97% (*n* = 11)
More than a day without problems	24.79% (*n* = 29)	62.07% (*n* = 36)
