# Supplementary material for: Media use among children with ASD: Perspectives and concerns of parents
Source: PLoS One. 2025 Oct 13;20(10):e0332504. doi: 10.1371/journal.pone.0332504 (PMC12517494; doi:10.1371/journal.pone.0332504)
Supplement: S12 Table — (PDF) [file pone.0332504.s018.pdf]

S12 Table. Necessity of media use for the child currently

Media use for the child	ASD (*n* = 107)	TD (*n* = 56)
not necessary	2.8% (*n* = 3)	16.07% (*n* = 9)
practical, but dispensable	20.56% (*n* = 22)	57.14% (*n* = 32)
indispensable	76.64% (*n* = 82)	26.79% (*n* = 15)
