# Supplementary material for: Media use among children with ASD: Perspectives and concerns of parents
Source: PLoS One. 2025 Oct 13;20(10):e0332504. doi: 10.1371/journal.pone.0332504 (PMC12517494; doi:10.1371/journal.pone.0332504)
Supplement: S13 Table — (PDF) [file pone.0332504.s019.pdf]

S13 Table. Code booklet for the Excel file containing the study data

**Variable name**	**Description**	**Coding**
id	ID for participating person	
Group	Group (ASD or TD)	ASD = child with ASD<br>TD = child with TD
HaNu[Geraet1][1]	Availability of PC/Laptop in the home	0 = not present in the household<br>1 = present in the household
HaNu[Geraet1][2]	Frequency of use: PC/Laptop	0 = never<br>1 = a few times a month<br>2 = several times a week<br>3 = daily
HaNu[Geraet2][1]	Availability of Tablet in the home	0 = not present in the household<br>1 = present in the household
HaNu[Geraet2][2]	Frequency of use: Tablet	0 = never<br>1 = a few times a month<br>2 = several times a week<br>3 = daily
HaNu[Geraet3][1]	Availability of Smartphone in the home	0 = not present in the household<br>1 = present in the household
HaNu[Geraet3][2]	Frequency of use: Smartphone	0 = never<br>1 = a few times a month<br>2 = several times a week<br>3 = daily
HaNu[Geraet4][1]	Availability of Gaming console in the home	0 = not present in the household<br>1 = present in the household
HaNu[Geraet4][2]	Frequency of use: Gaming console	0 = never<br>1 = a few times a month<br>2 = several times a week<br>3 = daily
HaNu[Geraet5][1]	Availability of Television in the home	0 = not present in the household<br>1 = present in the household
HaNu[Geraet5][2]	Frequency of use: Television	0 = never<br>1 = a few times a month<br>2 = several times a week<br>3 = daily

Variable name	Description	Coding
HaNu[Geraet6][1]	Availability of Radio/Music recorder (Tonieboxen) in the home	0 = not present in the household<br>1 = present in the household
HaNu[Geraet6][2]	Frequency of use: Radio/Music recorder (Tonieboxen	0 = never<br>1 = a few times a month<br>2 = several times a week<br>3 = daily
HaNu[Geraet7][1]	Availability of digital assistants (Alexa) in the home	0 = not present in the household<br>1 = present in the household
HaNu[Geraet7][2]	Frequency of use: Digital assistants (Alexa)	0 = never<br>1 = a few times a month<br>2 = several times a week<br>3 = daily
HaNu[Geraet8][1]	Availability of Smart-Toys in the home	0 = not present in the household<br>1 = present in the household
HaNu[Geraet8][2]	Frequency of use: Smart-Toys	0 = never<br>1 = a few times a month<br>2 = several times a week<br>3 = daily
EigGer[SQ001]	Own property of the child: Smartphone	0 = does not have its own<br>1 = does have its own
EigGer[SQ002]	Own property of the child: Computer/Laptop	0 = does not have its own<br>1 = does have its own
EigGer[SQ003]	Own property of the child: Tablet/IPad	0 = does not have its own<br>1 = does have its own
EigGer[SQ004]	Own property of the child: Television	0 = does not have its own<br>1 = does have its own
EigGer[SQ005]	Own property of the child: Gaming console	0 = does not have its own<br>1 = does have its own
KiZim	Digital devices available and usable in the children´s room	0 = no<br>1 = yes<br>2 = we only have a shared living space

Variable name	Description	Coding
Zeit[Geraet1][1]	Average daily time per week: PC/Laptop	0 = never<br>1 = up to 30 min<br>2 = 30 min to 2 hours<br>3 = 2 hours to 4 hours<br>4 = 4 hours and more
Zeit[Geraet1][2]	Average daily time per weekends/holidays: PC/Laptop	0 = never<br>1 = up to 30 min<br>2 = 30 min to 2 hours<br>3 = 2 hours to 4 hours<br>4 = 4 hours and more
Zeit[Geraet2][1]	Average daily time per week: Tablet	0 = never<br>1 = up to 30 min<br>2 = 30 min to 2 hours<br>3 = 2 hours to 4 hours<br>4 = 4 hours and more
Zeit[Geraet2][2]	Average daily time per weekends/holidays: Tablet	0 = never<br>1 = up to 30 min<br>2 = 30 min to 2 hours<br>3 = 2 hours to 4 hours<br>4 = 4 hours and more
Zeit[Geraet3][1]	Average daily time per week: Smartphone	0 = never<br>1 = up to 30 min<br>2 = 30 min to 2 hours<br>3 = 2 hours to 4 hours<br>4 = 4 hours and more
Zeit[Geraet3][2]	Average daily time per weekends/holidays: Smartphone	0 = never<br>1 = up to 30 min<br>2 = 30 min to 2 hours<br>3 = 2 hours to 4 hours<br>4 = 4 hours and more
Zeit[Geraet4][1]	Average daily time per week: Gaming console	0 = never<br>1 = up to 30 min<br>2 = 30 min to 2 hours<br>3 = 2 hours to 4 hours<br>4 = 4 hours and more
Zeit[Geraet4][2]	Average daily time per weekends/holidays: Gaming console	0 = never<br>1 = up to 30 min<br>2 = 30 min to 2 hours<br>3 = 2 hours to 4 hours<br>4 = 4 hours and more

Variable name	Description	Coding
Zeit[Geraet5][1]	Average daily time per week: Music recorder (including Tonieboxes, audio plays)	0 = never<br>1 = up to 30 min<br>2 = 30 min to 2 hours<br>3 = 2 hours to 4 hours<br>4 = 4 hours and more
Zeit[Geraet5][2]	Average daily time per weekends/holidays: Music recorder (including Tonieboxes, audio plays)	0 = never<br>1 = up to 30 min<br>2 = 30 min to 2 hours<br>3 = 2 hours to 4 hours<br>4 = 4 hours and more
Zeit[Geraet6][1]	Average daily time per week: Television (including streaming services such as Netflix, Disney+)	0 = never<br>1 = up to 30 min<br>2 = 30 min to 2 hours<br>3 = 2 hours to 4 hours<br>4 = 4 hours and more
Zeit[Geraet6][2]	Average daily time per weekends/holidays: Television (including streaming services such as Netflix, Disney+)	0 = never<br>1 = up to 30 min<br>2 = 30 min to 2 hours<br>3 = 2 hours to 4 hours<br>4 = 4 hours and more
Altreg	Indication of whether or not the child already uses digital media on a daily basis in german	
Altreg_eng	Indication of whether or not the child already uses digital media on a daily basis in english	
Altreg[other]	The age at which the child starts to use digital media on a daily basis.	
Altreg1	The age at which parents would first allow their children to use digital media every day.	
Altreg_code	Indication of whether or not the child already uses digital media on a daily basis	0 = My child does not use digital media every day yet<br>1 = My child uses digital media every day
Altreg_Zahl	Age of child's daily digital media consumption	
MeZeitTag[inWo_Stunde]	Maximum media time per day during the week (hour)	
MeZeitTag[inWo_Minute]	Maximum media time per day during the week (minute)	

Variable name	Description	Coding
MeZeitTag[WoEnd_Stunde]	Maximum media time per day during the weekend (hour)	
MeZeitTag[WoEnd_Minute]	Maximum media time per day during the weekend (minute)	
MeZeitTag_Wo_Minuten	Maximum media time per day during the week in minutes	
MeZeitTag_WoEnd_Minuten	Maximum media time per day during the weekend in minutes	
GruNut[Grund1]	What children do when they use media: entertainment games	0 = never<br>1 = a few times a month<br>2 = several times a week<br>3 = daily
GruNut[Grund2]	What children do when they use media: educational games	0 = never<br>1 = a few times a month<br>2 = several times a week<br>3 = daily
GruNut[Grund3]	What children do when they use media: view photos	0 = never<br>1 = a few times a month<br>2 = several times a week<br>3 = daily
GruNut[Grund4]	What children do when they use media: take photos	0 = never<br>1 = a few times a month<br>2 = several times a week<br>3 = daily
GruNut[Grund5]	What children do when they use media: listen to music/audio plays (including Tonieboxes)	0 = never<br>1 = a few times a month<br>2 = several times a week<br>3 = daily
GruNut[Grund6]	What children do when they use media: watch movies/videos	0 = never<br>1 = a few times a month<br>2 = several times a week<br>3 = daily
GruNut[Grund7]	What children do when they use media: programming	0 = never<br>1 = a few times a month<br>2 = several times a week<br>3 = daily
GruNut[Grund8]	What children do when they use media: chat/talk with others	0 = never<br>1 = a few times a month<br>2 = several times a week<br>3 = daily

Variable name	Description	Coding
MitbekNu	To what extent parents are aware of their child´s media use	0 = (almost) not at all<br>1 = partly<br>2 = completely
SituaGer[SQ001]	Situation in which meda are provided: To bridge waiting times	0 = never<br>1 = sometimes<br>2 = often<br>3 = regularly
SituaGer[SQ002]	Situation in which meda are provided: when my child is bored	0 = never<br>1 = sometimes<br>2 = often<br>3 = regularly
SituaGer[SQ003]	Situation in which meda are provided: to support learning	0 = never<br>1 = sometimes<br>2 = often<br>3 = regularly
SituaGer[SQ004]	Situation in which meda are provided: to have time for other things myself	0 = never<br>1 = sometimes<br>2 = often<br>3 = regularly
SituaGer[SQ005]	Situation in which meda are provided: as family time together	0 = never<br>1 = sometimes<br>2 = often<br>3 = regularly
SituaGer[SQ006]	Situation in which meda are provided: when my child is not feeling well	0 = never<br>1 = sometimes<br>2 = often<br>3 = regularly
SituaGer[SQ007]	Situation in which meda are provided: when my own stress limits are reached	0 = never<br>1 = sometimes<br>2 = often<br>3 = regularly
SituaGer[SQ008]	Situation in which meda are provided: To do household chores undisturbed	0 = never<br>1 = sometimes<br>2 = often<br>3 = regularly
SituaGer[SQ009]	Situation in which meda are provided: as a reward	0 = never<br>1 = sometimes<br>2 = often<br>3 = regularly

Variable name	Description	Coding
SituaGer_Code	Situation in which meda are provided: others in german	
SituaGer_Code_eng	Situation in which meda are provided: others in english	
Regeln	Rules for media use	0 = no<br>1 = yes
Aushalohn	How many hours can the child cope without media	0 = not at all<br>1 = 1-2 hours<br>2 = 3-4 hours<br>3 = 5-6 hours<br>4 = 7-12 hours<br>5 = a whole day<br>6 = without problems more than one day
NotweNu	How do you currently rate the necessity of media use for your child in german	
NotweNu_eng	How do you currently rate the necessity of media use for your child in english	
UmgangM5	My child is looking forward to using digital media (again).	1 = Never applies<br>to<br>10 = (almost) always true
UmgangI2	My child is so immersed in digital media that he/she forgets everything around him/her.	1 = Never applies<br>to<br>10 = (almost) always true
UmgangM3	My child prefers digital media to spending time with other people, e.g. parents, friends.	1 = Never applies<br>to<br>10 = (almost) always true
UmgangV3	My child spends too much time with digital media.	1 = Never applies<br>to<br>10 = (almost) always true
UmgangM4	My child doesn't sleep enough because of digital media.	1 = Never applies<br>to<br>10 = (almost) always true
UmgangI1	My child forgets something important because he/she have used digital media.	1 = Never applies<br>to<br>10 = (almost) always true
UmgangM6	My child completes task at home hastily so that he/she can use digital media earlier.	1 = Never applies<br>to<br>10 = (almost) always true

Variable name	Description	Coding
UmgangF3	Other people (e.g. family friends, grandparents, teachers) tell my child that he/she should use digital media less often.	1 = Never applies<br>to<br>10 = (almost) always true
UmgangM7	My child feels bad when they are not allowed to use digital devices.	1 = Never applies<br>to<br>10 = (almost) always true
UmgangS2	My child is confident in using digital media.	1 = Never applies<br>to<br>10 = (almost) always true
UmgangM1	My child finds it difficult to stop using digital media.	1 = Never applies<br>to<br>10 = (almost) always true
UmgangF4	Other people (e.g. other parent, friend, grandparents) tell me that my child should use digital media less often.	1 = Never applies<br>to<br>10 = (almost) always true
UmgangM2	My child continues to use digital media even though he/she is supposed to stop.	1 = Never applies<br>to<br>10 = (almost) always true
UmgangF2	My child's media time leads to conflicts in the the family.	1 = Never applies<br>to<br>10 = (almost) always true
UmgangM8	My child has to use digital devices for a long time andor frequently in order to feel good or relaxed again.	1 = Never applies<br>to<br>10 = (almost) always true
UmgangS4	I trust my child to be able to decide for himself/herself how much/how long he/she use digital media.	1 = Never applies<br>to<br>10 = (almost) always true
UmgangV1	My child's desire for digital media is often more pronounced than I think is good.	1 = Never applies<br>to<br>10 = (almost) always true
UmgangE2	I limit my child's media use.	1 = Never applies<br>to<br>10 = (almost) always true
UmgangV2	My child's desire for digital media is so overwhelming that I allow him/her to use it after all.	1 = Never applies<br>to<br>10 = (almost) always true

Variable name	Description	Coding
UmgangS3	I trust my child to be able to decide for himself/herself how much/how long he/she use digital media.	1 = Never applies<br>to<br>10 = (almost) always true
UmgangE1	I find it difficult to limit my child's media consumption.	1 = Never applies<br>to<br>10 = (almost) always true
UmgangF1	I avoid negative feeling of my child (e.g. boredom, anger, sagness, shutdowns) through media use.	1 = Never applies<br>to<br>10 = (almost) always true
UmgangE3	I am able to limit my child's media use	1 = Never applies<br>to<br>10 = (almost) always true
UmgangS1	I feel confident in dealing with digital media.	1 = Never applies<br>to<br>10 = (almost) always true
UmgangE2R	I limit my child's media use. - recoded	1 = (almost) always true<br>to<br>10 = Never applies
UmgangE3R	I am able to limit my child's media use. - recoded	1 = (almost) always true<br>to<br>10 = Never applies
SorgenS1	I am worried about my child's media use, that my child spend too much time with digital media.	1 = Disagree<br>to<br>10 = Totally agree
SorgenRW2	I am worried about my child's media use, that that my child is losing real-life friends.	1 = Disagree<br>to<br>10 = Totally agree
SorgenK1	I am worried about my child's media use, that my child is letting himself/herself go physically.	1 = Disagree<br>to<br>10 = Totally agree
SorgenS3	I am worried about my child's media use, that my child spends more time with digital devices than other children their age.	1 = Disagree<br>to<br>10 = Totally agree
SorgenK3	I am worried about my child's media use, that my child is developing aggressive behavior.	1 = Disagree<br>to<br>10 = Totally agree

Variable name	Description	Coding
SorgenO1	I am worried about my child's media use, that my child is being bullied online.	1 = Disagree<br>to<br>10 = Totally agree
SorgenK2	I am worried about my child's media use, that my child's eyes are getting worse.	1 = Disagree<br>to<br>10 = Totally agree
SorgenRW4	I am worried about my child's media use, that my child is losing touch with real world.	1 = Disagree<br>to<br>10 = Totally agree
SorgenS2	I am worried about my child's media use, that my child is becoming addicted.	1 = Disagree<br>to<br>10 = Totally agree
SorgenO2	I am worried about my child's media use, that my child gets involved with the wrong people online.	1 = Disagree<br>to<br>10 = Totally agree
SorgenK4	I am worried about my child's media use, that my child will develop an inpairment.	1 = Disagree<br>to<br>10 = Totally agree
SorgenRWL5	I am worried about my child's media use, that my child's social skills are declining.	1 = Disagree<br>to<br>10 = Totally agree
SorgenO3	I am worried about my child's media use, that my child reveals too much about himself/herself online.	1 = Disagree<br>to<br>10 = Totally agree
SorgenRW1	I am worried about my child's media use, that my child is losing interest in non-media activities.	1 = Disagree<br>to<br>10 = Totally agree
SorgenI3	I am worried about my child's media use, that I am not familiar enough with digital media.	1 = Disagree<br>to<br>10 = Totally agree
SorgenK5	I am worried about my child's media use, that my child is developing autistic behavior.	1 = Disagree<br>to<br>10 = Totally agree
SorgenRW3	I am worried about my child's media use, that my child is getting lost in the digital world.	1 = Disagree<br>to<br>10 = Totally agree
SorgenI1	I am worried about my child's media use, that I no longer have any control over my child's life.	1 = Disagree<br>to<br>10 = Totally agree

Variable name	Description	Coding
SorgenI4	I am worried about my child's media use, that I forbid my child to use digital media too much.	1 = Disagree<br>to<br>10 = Totally agree
SorgenU1	I am worried about my child's media use, because I don't receive any support in the use of digital media.	1 = Disagree<br>to<br>10 = Totally agree
SorgenI2	I am worried about my child's media use, that I am losing contact with my child.	1 = Disagree<br>to<br>10 = Totally agree
SorgenK6	I am worried about my child's media use, that my child is developing/has developed autism.	1 = Disagree<br>to<br>10 = Totally agree
SorgenI5	I am worried about my child's media use, that I am overconfident in my child's use of digital media.	1 = Disagree<br>to<br>10 = Totally agree
SorgenU2	I am worried about my child's media use, because I don't receive any support with my child's media education.	1 = Disagree<br>to<br>10 = Totally agree
SorgenI6	I am worried about my child's media use, that I give my child too much freedom.	1 = Disagree<br>to<br>10 = Totally agree
KAlter[Jahr]	Age of the child (year)	
KAlter[Monate]	Age of the child (month)	
KAlter_Gesamtmonate	Age of the child in month	
Kgesch_code	Gender of the child	0 = male<br>1 = female<br>2 = diverse
KGesch	Gender of the child in german	
KGesch_eng	Gender of the child in english	
KBeein	Child has a disability	0 = no<br>1 = yes
K_Beein_alle	Diagnosed impairments in the child in german	
K_Beein_alle_eng	Diagnosed impairments in the child in english	

Variable name	Description	Coding
KSprech	The child can communicate with other in an age-appropiate way.	0 = does not apply (no verbal language)<br>1 = Applies to a limited extent<br>2 = Applies
KGeschw	Whether or not the child has siblings.	0 = no siblings<br>1 = siblings
KGeschw[other]	Number of siblings of the child	
EBezug	Legal guardian who completed the questionnaire in german	
EBezug[other]	Legal guardian who completed the questionnaire in german	
EBezug_eng	completed the questionnaire in english	
EAlter	Age of the legal guardian	
EBeein	Legal guardian has a disability	0 = no<br>1 = yes
EBeein[other]	Diagnosed impairments in the legal guardian(s) in german	
EBeein[other]_eng	Diagnosed impairments in the legal guardian(s) in english	
EBildu[SQ001]	Highest educational qualification of the lega lguardian person 1 (e.g. mother)	0 = without high school diploma<br>1 = General Certificate of Education<br>2 = intermediate-secondary school-leaving certificate<br>3 = subject-related entrance qualification<br>4 = higher eduction entrance qualification (A-levels)<br>5 = University degree<br>6 = Doctorate

Variable name	Description	Coding
EBildu[SQ002]	Highest educational qualification of the legal guardian person 2 (e.g. father)	0 = without high school diploma<br>1 = General Certificate of Education<br>2 = intermediate-secondary school-leaving certificate<br>3 = subject-related entrance qualification<br>4 = higher eduction entrance qualification (A-levels)<br>5 = University degree<br>6 = Doctorate
